# Intermittent hypoxic conditioning as a novel strategy for obesity treatment: current evidence and future perspectives

**DOI:** 10.3389/fphys.2026.1842034

**Published:** 2026-06-26

**Authors:** Yazhi Kang, Jianfei Wen, Li Xi, Yufei Qi, Xiaoyan Chen, Na Li

**Affiliations:** 1 College of Sports Science, Hefei Normal University, Hefei, China; 2 Key Laboratory of Philosophy and Social Science of Anhui Province on Adolescent Mental Health and Crisis Intelligence Intervention, Hefei Normal University, Hefei, China; 3 School of Physical Education, Hefei Normal University, Hefei, China; 4 Department of Physical Education and Research, Central South University, Changsha, China; 5 Scientific Research Center, Anhui Provincial Sports Science and Technology Research Institute, Hefei, China

**Keywords:** exercise, intermittent hypoxic conditioning, mechanisms, obesity, therapeutic potential

## Abstract

Obesity is a metabolic disorder characterized by excessive accumulation of adipose tissue and is a recognized risk factor for numerous chronic diseases. Effective non-pharmacological management of obesity is essential for reducing obesity-related health risks. Hypoxia, defined as insufficient oxygen supply to tissues and cells, is associated with pathological and pathophysiological conditions. In recent years, intermittent hypoxic conditioning (IHC), a potential non−pharmacological intervention under controlled hypoxic conditions, has gained increasing popularity. Recent studies suggest potential benefits of IHC in improving disease-related outcomes. A growing body of research supports its feasibility as an intervention for obesity, highlighting its emerging potential in the treatment of this condition. This review critically discusses the effects of IHC on obesity, with a focus on both animal and human evidence, underlying mechanisms, and safety considerations. By synthesizing current findings, it aims to clarify the state of knowledge and research limitations, and to provide an objective assessment of the applicability of IHC in obesity management.

## 1 Introduction

Obesity, defined by the World Health Organization (WHO) as ‘‘abnormal or excessive fat accumulation that presents a risk to health’’, has emerged as a critical global public health challenge (Ruban et al., 2019; Bray, 2025). This chronic disease is strongly associated with elevated morbidity and mortality from various comorbidities, significantly reducing quality of life and imposing substantial disease burdens worldwide (Peters et al., 2018; Piché et al., 2020; Koskinas et al., 2024). Epidemiological data indicate that obesity prevalence has risen dramatically across all age groups worldwide over recent decades (Blüher, 2019). Although conventional interventions such as lifestyle modification, anti-obesity medications (AOMs), and bariatric surgery are widely available, their long-term effectiveness is limited by a range of factors, such as poor adherence, side effects, high costs, and invasiveness (Perdomo et al., 2023; Yanovski and Yanovski, 2024). These limitations have prompted the search for alternative strategies that combine meaningful efficacy with improved safety, lower costs, and greater convenience for patients.

One emerging approach that has attracted increasing attention is hypoxia-based intervention. Hypoxia is known to exert beneficial effects on human health by triggering adaptive physiological responses. In the 1980s, Russian scientists proposed the concept that inhalation of controlled oxygen-reduced gas at atmospheric pressure could produce physiological responses comparable to high-altitude or hypobaric chamber exposure (Elena et al., 2022). This concept laid the foundation for the development of various hypoxia-based intervention modalities. Among these, intermittent hypoxic conditioning (IHC) refers to alternating cyclic exposure between hypoxic conditions and normoxia or hyperoxia, with main modalities including intermittent hypoxic exposure (IHE, hypoxia-normoxia at rest), intermittent hypoxic training (IHT, hypoxia-normoxia with exercise), intermittent hypoxic-hyperoxia exposure (IHHE, hypoxia-hyperoxia at rest), and intermittent hypoxic-hyperoxia training (IHHT, hypoxia-hyperoxia with exercise) (Yuan et al., 2022; Zhang et al., 2024). Although these modalities differ in exercise involvement and reoxygenation type, they all rely on intermittent hypoxia to trigger adaptive responses. Importantly, accumulating evidence suggests that IHC has a role in managing a range of conditions, including neurological, respiratory, cardiovascular and metabolic disorders (Zhang et al., 2023). These collective findings position IHC as an emerging intervention for health promotion and disease prevention.

In the context of obesity, IHC has been explored as a potential non-pharmacological intervention, owing to its capacity to induce adaptive responses involving metabolic remodeling, antioxidant defense, and anti-inflammatory pathways (Burtscher et al., 2024). Importantly, this controlled, dose-defined hypoxic intervention differs fundamentally from the pathological intermittent hypoxia characteristic of conditions such as obstructive sleep apnea, which is driven by chronic uncontrolled nocturnal desaturation-reoxygenation cycles that promote oxidative stress and cardiovascular damage (Lv et al., 2023). Notably, whether IHC produces beneficial or harmful outcomes is determined by multiple factors, including the baseline physiological status of the obese individual and the design of the IHC protocol. On the one hand, obesity itself is characterized by physiological alterations, including respiratory impairment, exercise intolerance, and adipose tissue hypoxia, which may reduce the capacity to tolerate hypoxic and exercise stress and increase the risk of adverse responses (Busebee et al., 2023). On the other hand, the hypoxic dose and exercise protocol critically determine the magnitude of the physiological stimulus. Controlled, moderate hypoxic exposures can promote physiological adaptation, whereas excessive or prolonged hypoxia may shift adaptation toward maladaptation (Burtscher et al., 2024). These interacting factors may help explain the conflicting results in the literature. For instance, some studies have reported favorable effects of IHC on body weight, visceral fat mass, and vascular compliance in certain obese populations (Bagińska et al., 2024; Jiao et al., 2024). However, others have found no clear superiority of IHC over normoxic exercise (Abdallah et al., 2022). These observations emphasize the importance of evaluating both the efficacy and safety of IHC to critically assess its role in obesity management.

Previous reviews have focused primarily on the mechanisms underlying exercise-based hypoxic interventions in obesity, with a relatively narrow scope of IHC modalities, and were based largely on earlier studies without incorporating recent advances in the field (Aritz et al., 2011; Tee et al., 2023a). Furthermore, a critical aspect, the safety of IHC when applied to obesity, has not been specifically addressed in these reviews. In light of these limitations, the present review focuses on three key issues. First, it synthesizes recent animal and clinical studies to provide an updated and critical evaluation of the current evidence, covering both therapeutic outcomes and underlying mechanisms. Second, it devotes specific attention to the safety of IHC in obesity, emphasizing individualized monitoring and treatment strategies. Third, it systematically discusses the limitations of existing studies. Through these efforts, this review aims to offer a more comprehensive assessment of IHC in obesity management.

## 2 Survey methodology

A literature search was conducted in PubMed (https://pubmed.ncbi.nlm.nih.gov/) and Web of Science (https://www.webofscience.com/) databases. The search was not restricted by publication date, but priority was given to studies published within the last 10 years. The search utilized keywords and phrases including “intermittent hypoxic conditioning”, “intermittent hypoxic training”, “intermittent hypoxic exposure”, “intermittent hypoxia”, “hypoxic training”, “obesity”, “obese”, “overweight”, “body weight”, and “mechanisms”. The inclusion criteria were: (1) studies focusing on IHC; (2) basic science or clinical research investigating IHC for obesity and its underlying mechanisms; (3) original articles and reviews. The exclusion criteria were: (1) unavailability of the full text; (2) duplicate or redundant publications; (3) non-English publications; (4) studies irrelevant to the research theme. The screening process involved two phases: an initial assessment based on titles, followed by a review of abstracts and keywords. Additional relevant studies were identified through screening of reference lists of included articles.

## 3 Obesity: pathophysiological features and therapeutic landscape

**Figure 1:**
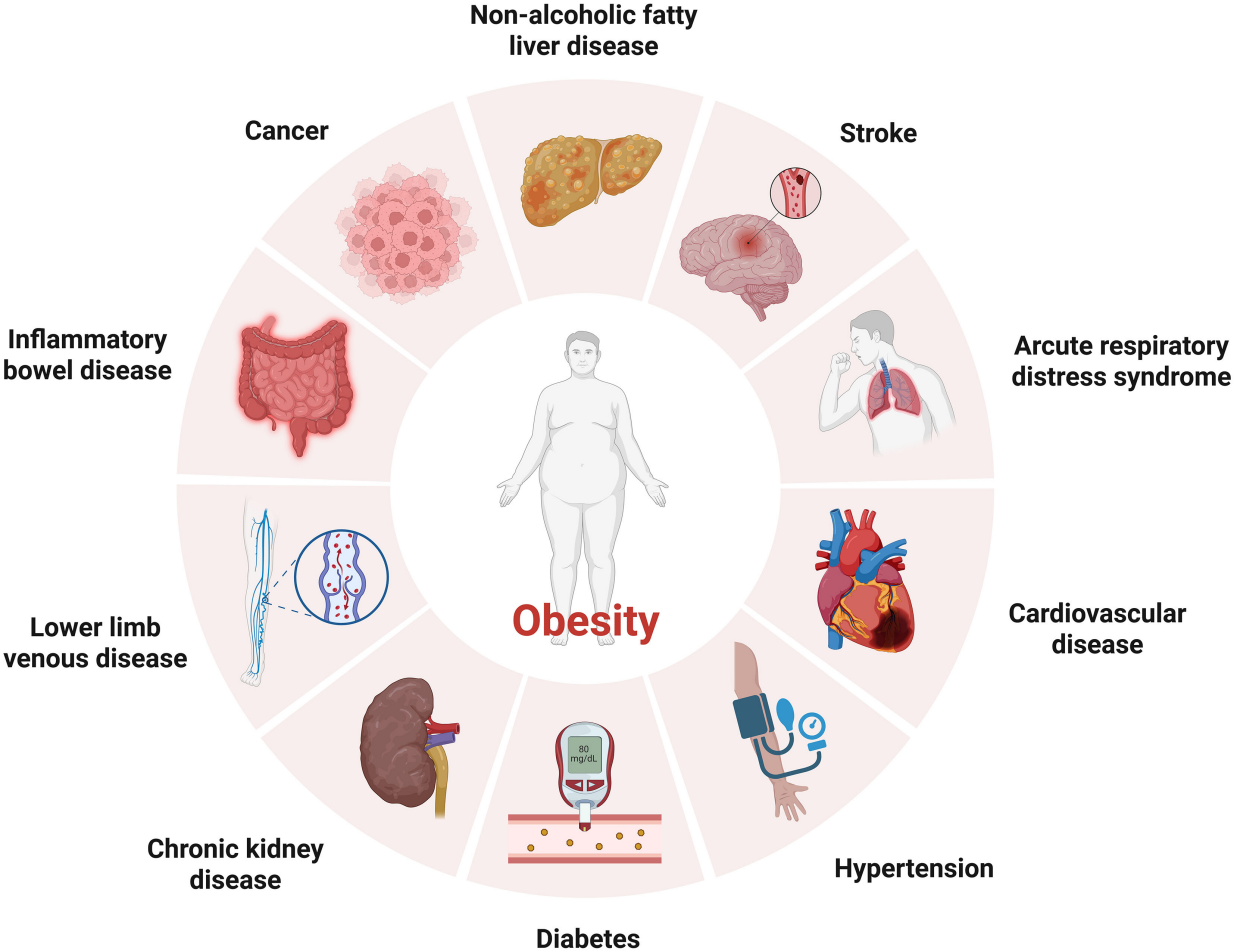
Common obesity-related comorbidities.

Obesity, defined by the WHO as a BMI ≥ 30 kg/m^2^, has emerged as a major global public health challenge (Consultation, 2000). Epidemiological data indicate that obesity prevalence has risen dramatically across all age groups worldwide over recent decades, driven by unhealthy dietary habits, decreased physical activity, chronic stress exposure, and environmental influences (Conway and Rene, 2004; Jebeile et al., 2022; Shams et al., 2022; Chandrasekaran and Weiskirchen, 2024). Obesity is a major risk factor for the development of several diseases, including type 2 diabetes, hypertension, and cardiovascular disease, and is increasingly recognized as a complex, multifactorial disease entity rather than simply a risk factor for associated comorbidities (**Figure 1**) (Piché et al., 2020; Yao et al., 2023; Chandrasekaran and Weiskirchen, 2024). This section describes the key physiological alterations associated with obesity and reviews current treatment strategies and their limitations. Understanding these aspects is fundamental to addressing the challenges of obesity management.

### 3.1 Obesity-related physiological alterations

Obesity is characterized by several physiological alterations that are directly relevant to interventions involving hypoxia or exercise, including respiratory impairment, exercise intolerance, impaired oxygen utilization, and adipose tissue hypoxia (Lempesis et al., 2020; Busebee et al., 2023).

Obesity impairs both resting and exercise-related respiratory function, increasing the risk of adverse events during hypoxic exposure. Specifically, excess adiposity in the chest and abdomen increases intra-abdominal and intrathoracic pressure, altering upper airway structure, reducing pulmonary function, and contributing to ventilatory abnormalities (Busebee et al., 2023). Meanwhile, obesity is associated with heightened ventilatory demand, reduced expiratory reserve volume, reduced respiratory muscle efficiency, and compromised respiratory compliance (Krishnan et al., 2006; Akshay, 2009). Together with mechanical ventilatory constraints, these changes increase inspiratory muscle activity and load, making them more susceptible to exercise-induced respiratory muscle fatigue and rapid desaturation under hypoxic conditions (Michael and Hans-Joachim, 2012).

In terms of exercise function, increased adipose mass imposes mechanical stress on musculoskeletal structures, altering skeletal alignment and increasing mechanical load, which compromises exercise capacity and raises the risk of injury (Busebee et al., 2023). At the same time, skeletal muscle oxidative capacity is reduced, as evidenced by slower oxygen uptake kinetics and impaired mitochondrial respiratory efficiency, and microvascular dysfunction further limits oxygen delivery to active muscles, together contributing to impaired oxygen utilization and reduced exercise tolerance (Inostroza-Mondaca et al., 2025). These pre-existing deficits may amplify the physiological strain imposed by hypoxic or exercise interventions, an issue that warrants careful consideration when applying such approaches in this population.

Adipose tissue dysfunction contributes to impaired glucose and lipid metabolism in obese individuals. In animal models, reduced capillary density and blood flow in adipose tissue can lead to regional hypoxia that drives inflammation and insulin resistance (Lempesis et al., 2020; Lempesis et al., 2022). However, findings in humans are more complex. Mitochondrial dysfunction in obese adipose tissue may reduce oxygen consumption, and some studies have even reported paradoxically elevated oxygen partial pressure in the adipose tissue of obese individuals (Lempesis et al., 2020; Lempesis et al., 2022). These observations suggest that adipose tissue oxygenation is altered in obesity, but the direction of this alteration may depend on the balance between oxygen supply and consumption. Regardless, it is clear that the adipose tissue microenvironment in obesity is characterized by disrupted oxygen homeostasis. Whether exposure to controlled, intermittent hypoxia improves or exacerbates this disruption remains an open question, highlighting the importance of careful safety evaluation when applying IHC in this population.

### 3.2 Current treatment strategies for obesity

**Figure 2:**
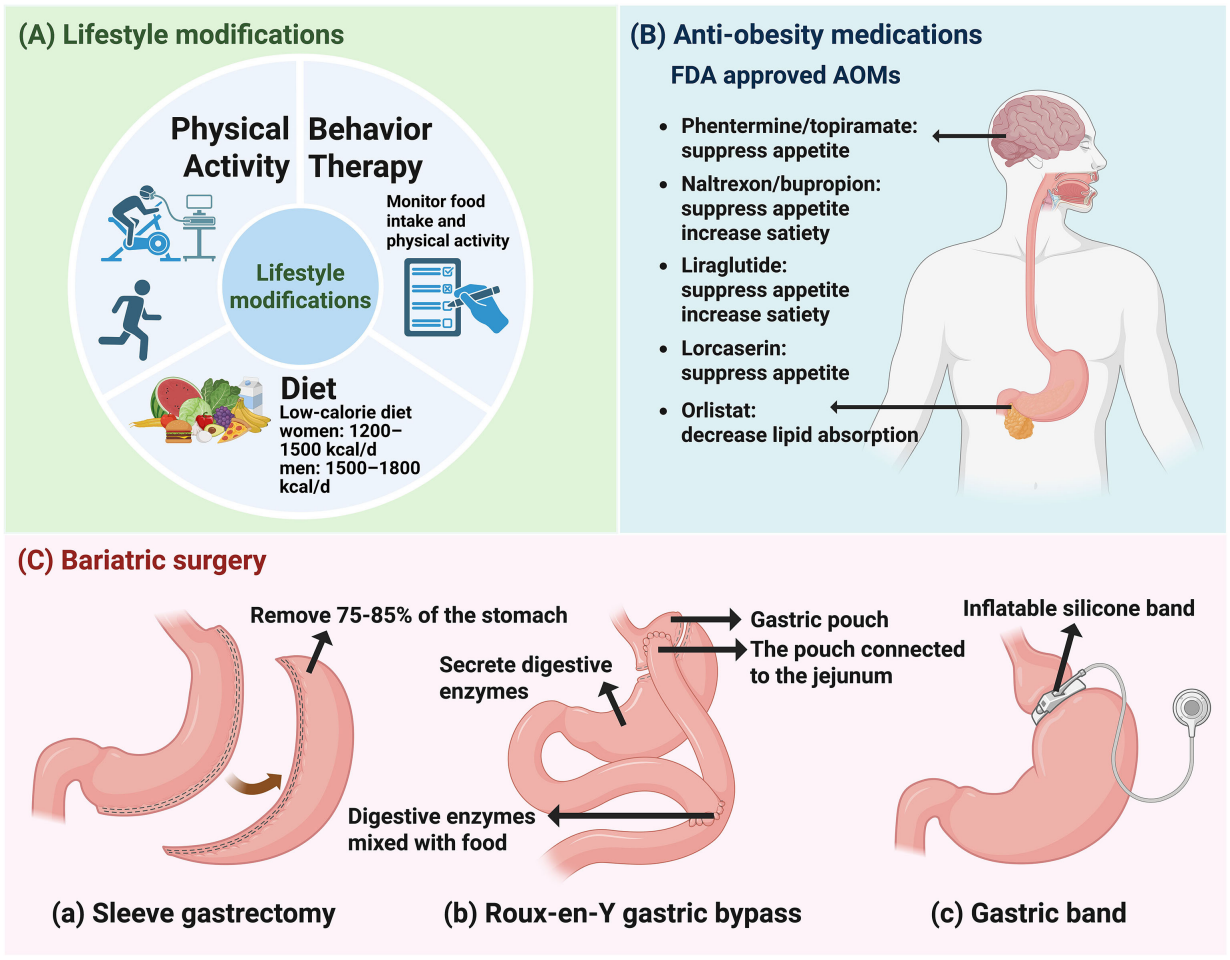
Current treatment strategies for obesity. **(A)** Lifestyle modifications. **(B)** AOMs. **(C)** Bariatric surgery: **(a)** Sleeve gastrectomy: longitudinal resection of approximately 75-85% of the stomach along the greater curvature, extending from the antrum to the fundus. **(b)** The Roux-en-Y gastric bypass: a restrictive gastric pouch (20–30 mL) is created to limit the passage of food. The small intestine is separated at the jejunum and the distal end of the intestine is connected to the gastric pouch. Food in the gastric pouch passes directly into the jejunum, which can limit food absorption. **(c)** Gastric band: a silicon inflatable gastric band is surgically positioned around the proximal stomach to create a small gastric pouch and restrict food intake.

The clinical weight reduction target for obese adults is usually to reduce total body weight by 5-10% to achieve meaningful health improvements (Wadden et al., 2020). As shown in **Figure 2**, evidence-based obesity management relies on three primary therapeutic approaches: lifestyle changes, AOMs, and bariatric surgery (Elmaleh-Sachs et al., 2023).

#### 3.2.1 Lifestyle modifications

Lifestyle modifications integrate dietary restrictions, physical activity, and behavioral therapy for weight reduction, and can achieve a 5% weight reduction over 24 months while lowering the risk of obesity-related complications, particularly type 2 diabetes and cardiovascular disease (Wadden et al., 2020; Kheniser et al., 2021; Beshir et al., 2023). Current guidelines recommend caloric restriction of 1200–1500 kcal/d for women and 1500–1800 kcal/d for men to achieve safe and effective weight loss (Jensen et al., 2013). Successful weight management also requires at least 175 minutes of intensive physical activity per week, yet long-term adherence to such regimens remains a major challenge (Olateju et al., 2023). Digital health technologies, including internet-based platforms and smartphone applications for real-time monitoring of dietary patterns, activity levels, and body weight, may help improve adherence (Wadden et al., 2020). Despite these measures, lifestyle modifications alone often fail to achieve or maintain long-term weight reduction, and current guidelines strongly advocate combining pharmacotherapy with lifestyle modifications for overweight individuals (Tak and Lee, 2021).

#### 3.2.2 AOMs

When lifestyle interventions are insufficient, pharmacotherapy represents an alternative approach (Nicolucci and Maffeis, 2022). To date, FDA-approved AOMs for chronic weight management include phentermine/topiramate, naltrexone/bupropion, liraglutide, lorcaserin, and orlistat (Beshir et al., 2023). These agents are less effective than bariatric surgery, but can provide up to 5% additive weight loss compared to lifestyle changes alone (Bray and Ryan, 2021). However, pharmacologic treatment of obesity has non-negligible limitations. First, notable safety issues concerns exist, including gastrointestinal symptoms (nausea, diarrhea, constipation), cardiovascular effects (elevated heart rate), and metabolic complications (Beshir et al., 2023; Chakhtoura et al., 2023). Second, the substantial financial burden poses challenges to medication accessibility, with annual costs frequently exceeding several thousand dollars and inconsistent insurance coverage, leading many patients to face financial barriers (Xue et al., 2023). In addition, AOMs are restricted to patients with a BMI ≥ 30 kg/m^2^ or a BMI ≥ 27 kg/m^2^ with weight-related comorbidities, and are therefore not suitable for all obese individuals (Chakhtoura et al., 2023).

#### 3.2.3 Bariatric surgery

For patients with severe obesity (BMI ≥ 40 kg/m^2^ or BMI ≥ 35 kg/m^2^ with comorbidities), when behavioral and pharmacological treatments fail to achieve sustained weight loss, bariatric surgery is an effective option (Jensen et al., 2013; Wolfe et al., 2016). As shown in **Figure 2**, the three primary procedures are sleeve gastrectomy (removal of 75-85% of the stomach), Roux-en-Y gastric bypass (creation of a small gastric pouch connected to the jejunum), and adjustable gastric band (contraction of the size of the gastric pouch by an adjustable gastric band) (Coutant et al., 2017; Fink et al., 2022; Eisenberg et al., 2023). Sleeve gastrectomy and Roux-en-Y gastric bypass have shown remarkable effectiveness, with sustained excess weight loss of 27-69% maintained for ≥ 10 years post-operation (Fink et al., 2022). Despite its superior efficacy, bariatric surgery has notable limitations. It is associated with complications such as micronutrient deficiencies and dumping syndrome, and some patients experience significant weight regain post−surgery (Fink et al., 2022; Courcoulas et al., 2023; Noria et al., 2023). Therefore, it requires careful patient selection and lifelong monitoring and is not a definitive solution for all individuals with severe obesity.

### 3.3 Current challenges and the promise of IHC

As noted above, the long-term success of conventional obesity interventions is often limited by poor long-term adherence to lifestyle changes, safety concerns and costs with AOMs, and invasiveness and the risk of complications with bariatric surgery. Moreover, access to these interventions remains limited in most regions, and weight regain following initial success is common (Jebeile et al., 2022). These limitations have motivated the exploration of alternative strategies. Among them, IHC has attracted increasing attention, owing to its potential to trigger adaptive responses through controlled intermittent hypoxic exposure, with or without exercise (Burtscher et al., 2024). In particular, IHC modalities that combine physical activity (IHT and IHHT) have been associated with additional metabolic and cardiovascular adaptations beyond those of conventional exercise, thereby providing superior weight loss benefits. For individuals unable to exercise, passive hypoxic exposure (IHE and IHHE) has shown metabolic benefits, which further broadens the applicability of IHC across different patient populations. The following sections introduce the basic concepts of IHC and critically examine the evidence for its application in obesity management.

## 4 IHC and human health

### 4.1 The basic concepts of IHC

IHC is a protocol involving alternating cyclic exposure to hypoxic conditions and normoxia (or hyperoxia), which facilitates physiological adaptation to reduced oxygen availability (Zhang et al., 2023). This concept originated from Russian researchers in the 1980s, who proposed that high-altitude acclimatization could be simulated through controlled inhalation of hypoxic gas mixtures at normal atmospheric pressure (Elena et al., 2022). To date, IHC has evolved into several distinct forms, including IHE (hypoxia-normoxia at rest), IHT (hypoxia-normoxia with exercise), IHHE (hypoxia-hyperoxia at rest), and IHHT (hypoxia-hyperoxia with exercise) (Yuan et al., 2022; Zhang et al., 2024). In IHC, inspired oxygen fraction (FiO_2_) is usually maintained between 9% and 15% (Kambič et al., 2025). Intermittent hypoxia-hyperoxia (IHH) is a variant of the intermittent hypoxia (IH) protocol, in which moderate hyperoxia (FiO_2_ = 30-40%) replaces the normoxic phase (Yuan et al., 2022). Currently, IHC is being explored for its applications across both healthy and diseased populations. On the one hand, IHC has become a popular training strategy for athletes to enhance athletic performance at sea-level altitudes. On the other hand, IHC has shown possible applications in multiple pathological conditions, including neurological, respiratory, cardiovascular, and metabolic disorders (Zhang et al., 2023).

**Figure 3:**
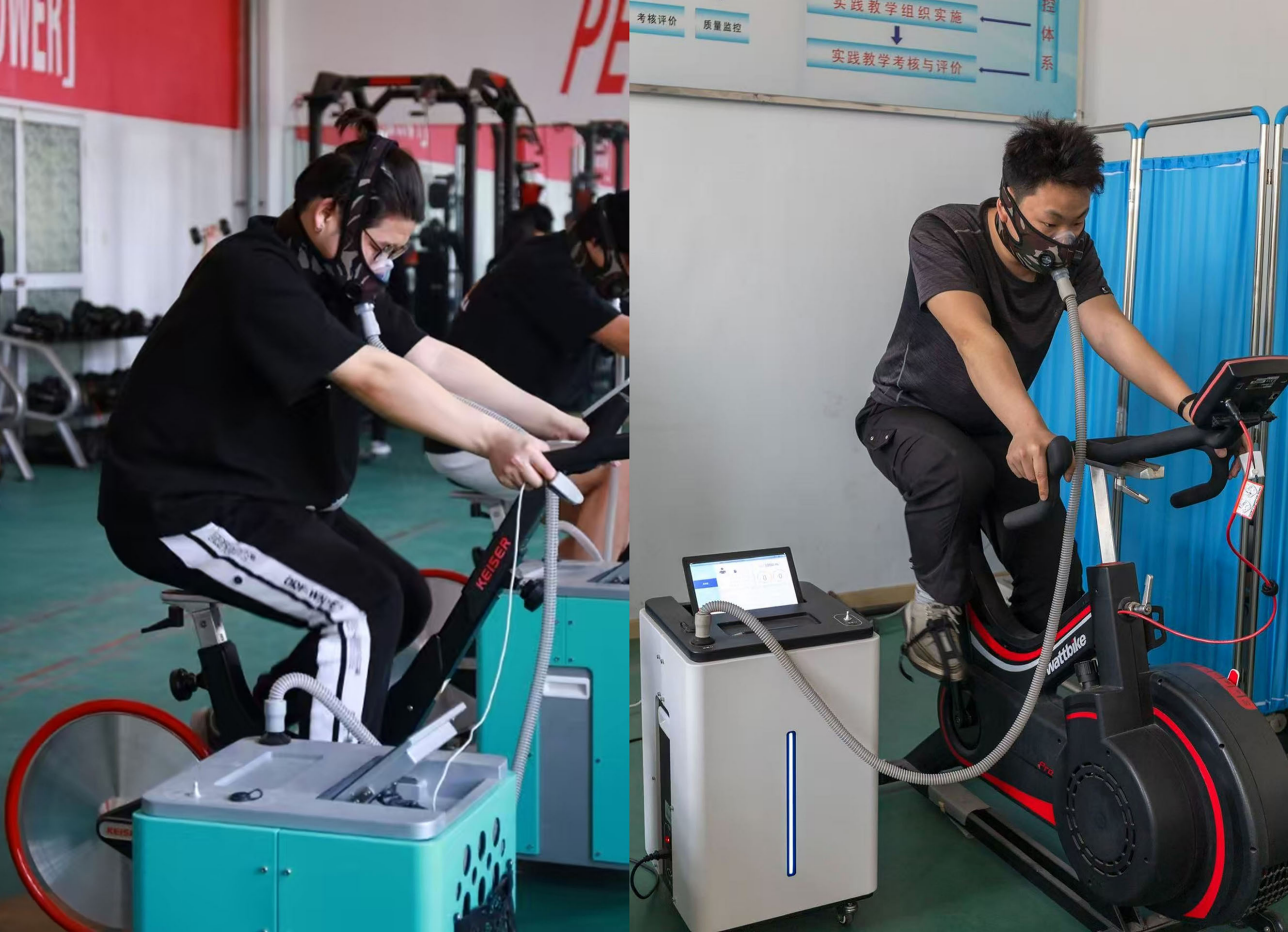
IHC (face mask method) for exercise intervention in humans.

Of course, the practical implementation of IHC protocols requires specialized equipment. Currently available options include hypobaric chambers, autohypoxicators, normobaric hypoxia rooms, tunnels, or tents, and hypoxic training mask systems (Serebrovskaya and Xi, 2016; Zhang et al., 2023). Of these, the mask system, consisting of a specialized face mask integrated with a gas flow circuit, is widely used in IHC for its convenience, efficacy, and cost-effectiveness (**Figure 3**).

### 4.2 Regimes of IHC

To date, no universally accepted IHC protocol exists, and regimens used in human practice vary widely in key parameters, particularly in exercise and hypoxic components (Zhang et al., 2023). Exercise parameters include exercise mode (e.g., repeated sprints or resistance training), as well as exercise intensity, duration, and frequency. Hypoxic parameters include intensity (e.g., FiO_2_ or simulated altitude), along with stimulus duration, cycles per session, intervention frequency, and total period. As a result, current evidence on IHC shows heterogeneity. For example, studies have found that regimens employing moderate hypoxia (9-16% O_2_) with low cycle frequency (3–15 episodes/day) are generally associated with positive outcomes (Navarrete-Opazo and Mitchell, 2014). In contrast, exposure to severe hypoxia (2-8% O_2_) at a high daily frequency (48–2400 episodes/day) tends to promote pathological changes (Navarrete-Opazo and Mitchell, 2014). Moreover, several IHC protocols using FiO_2_ in the range of 13-17% have reported protective effects in obese patients (P et al., 2015; Alba et al., 2018c; Jiao et al., 2024). Conversely, Serebrovska et al. found that treatment protocols with 13-15% FiO_2_ did not demonstrate clear beneficial effects, whereas a 2–3 week regimen of 12-10% FiO_2_ was associated with improved fitness and changes in disease-related outcomes without adverse consequences (T et al., 2016). This apparent contradiction implies that IHC intervention protocols (e.g., exercise and hypoxic parameters), participant characteristics (e.g., physical activity, nutritional status), and methodological factors (e.g., sample size, outcome measures) all likely contribute to the heterogeneity of reported outcomes. Therefore, IHC outcomes should be drawn on a case-by-case basis rather than overgeneralized. Accordingly, the development of personalized IHC protocols is essential to ensure optimal therapeutic outcomes. To ensure safety during IHC, we also recommend establishing a practical monitoring framework for IHC, including continuous peripheral oxygen saturation (SpO_2_) and heart rate tracking, blood pressure measurement (when appropriate), assessment of dyspnea, breathing discomfort, and perceived exertion using standardized scales. In addition, predefined stopping criteria should be established to terminate the IHC session when necessary. These measures will help ensure safety and optimize therapeutic outcomes across diverse populations.

### 4.3 Health benefits of IHC

IHC has been shown to exert protective effects on different tissue systems and is under investigation as an emerging approach for various human diseases (Zhang et al., 2023). Studies on cardiovascular disease reveal the role of IHC in improving cardiac resistance to ischemia-reperfusion stress, evidenced by a reduction in myocardial infarction and ventricular arrhythmias, and an improvement in contractile function and coronary blood flow (Mallet et al., 2018). Systematic research has also confirmed the cardiovascular benefits of IHC, demonstrating that it can improve exercise capacity and reduce arrhythmias in patients with coronary artery disease, while producing sustained antihypertensive effects in essential hypertension cases (Glazachev et al., 2021). Besides, emerging evidence suggests that IHC also has neuroprotective properties, especially in stroke management (Yuan et al., 2022). This intervention can benefit the nervous system by enhancing the resistance of the brain to acute ischemic-hypoxic injury and facilitating neurological repair after injury (Yuan et al., 2022).

Beyond these applications, research has also revealed the possible role of different IHC modalities in modulating metabolic health. Van Meijel et al. reported that mild IHE promoted metabolic adaptations in overweight/obese male subjects, including enhanced glycolytic metabolism and optimized tissue oxygenation (van Meijel et al., 2021). In the case of IHT, its metabolic modulatory effect appears to be mediated through the Sirtuin 1 (SIRT1)-dependent pathway, which contributes to the reduction of visceral fat mass in overweight/obese patients (Jiao et al., 2024). A prospective randomized controlled trial supported the capacity of IHHE to attenuate systemic inflammation and improve the lipid profile (total cholesterol (TC), low-density lipoprotein (LDL), and triglycerides) in patients with metabolic syndrome (Afina et al., 2021). Similarly, a nonrandomized controlled before-and-after trial in cardiac patients evaluated the effects of IHHE and found that repeated hypoxia (10-12% O_2_) and hyperoxia (30-35% O_2_) exposures improved both cardiometabolic and lipid profiles (TC, LDL, and the atherogenic index) (Glazachev et al., 2017). Collectively, these findings reflect that hypoxia-based interventions appear to have health-promoting potential, but effects may differ by modality, dose, and population.

## 5 IHC and obesity

Among the various conditions potentially affected by IHC, obesity has received particular attention, with research exploring its effects on both obesity and related complications.

### 5.1 Anti-obesity effects of IHC

#### 5.1.1 Animal research

Animal studies have provided preliminary evidence supporting the anti-obesity effects of IHC (**Table 1**). In obese female mice, 40 days of passive IHE (8 sessions/day, 15 min per session at 5 min intervals) markedly reduced body weight, blood glucose, and blood cholesterol, and prevented hepatic steatosis, an effect associated with regulation of the leptin/leptin receptor signaling pathway (Ling et al., 2008). Another study investigating the metabolic effects of IHE reported that 28 days of IHE (6 h/day at 5000 m simulated altitude) improved iron metabolism disorder in diet-induced obese rats, possibly via down-regulation of hepcidin (Cui et al., 2020). Furthermore, combining IHE (5 h/day, 33 days at 6000 m simulated altitude) with subsequent endurance training under normoxic conditions (1 h/day, 5 weeks) in adult male rats significantly reduced body weight, food intake, and oxygen consumption, highlighting that IHE combined with endurance training could be a useful tool for the treatment of overweight and obesity (Ignacio et al., 2018). These preliminary findings from animal models have prompted subsequent clinical studies to further evaluate the possible role of IHC in human populations.

**Table 1:** Preclinical evidence of IHC in obesity^*^.

No.	Animal	IHC modality	Hypoxic protocol	Equipment	Outcomes	Mechanism	Ref.
1	Obese female mice	IHE	PaO2 = 0.014 MPa, 40 days, 8 sessions/day (15 min/session, 5 min intervals)	Vacuum hypoxic box	Reduced body weight, blood glucose, blood cholesterol, and hepatic adipocyte distribution	Regulate leptin/leptin receptor signaling pathway	(Ling et al., 2008)
2	Obese male rats	IHE	6 h/day hypobaric hypoxia for 28 days, simulated 5000 m altitude	Hypobaric chamber	Improve iron metabolism	Down-regulation of hepcidin by inhibiting IL-6/JAK2/STAT3 pathway and activating Epo/STAT5/ERFE pathway	(Cui et al., 2020)

^*^ IHC, intermittent hypoxic conditioning; IHE, intermittent hypoxic exposure; PaO_2_, partial pressure of oxygen.

#### 5.1.2 Clinical research

Published studies have elucidated that alterations in oxygen supply can affect human body fat metabolism (Chia-Hua and Brennan, 2016). In particular, it has been found that the body fat of athletes shows marked decreases under hypoxic conditions, implying the possible application of hypoxic exercise in obesity interventions (Chia-Hua and Brennan, 2016). Already in 2017, a study conducted on healthy male participants found that normobaric hypoxic training protocols exerted beneficial effects on energy metabolism. The research revealed that the additional metabolic stress induced by hypoxia exposure markedly increased post-exercise lipid oxidation rates while showing a tendency to suppress carbohydrate oxidation (Liam and Fabien, 2017). Collectively, these findings support the metabolic benefits of hypoxic exercise. However, both studies involved healthy populations, direct evidence in obese individuals of different ages, sexes, and metabolic conditions require further investigation.

Currently, research on the therapeutic strategies for obesity has increasingly focused on IHC (**Table 2**). For overweight and obese women, several studies provided evidence that high-intensity interval training under normobaric hypoxia (12 weeks of cycle ergometer, 3 sessions/week at 2500 m simulated altitude) is more beneficial for decreasing body fat content, increasing muscle mass, and improving cardiometabolic risk markers than exercising under normoxia (Alba et al., 2018c; Alba et al., 2018b; Alba et al., 2019). In these studies, the IHT protocol strictly matched exercise intensity and workload between hypoxic (FiO_2_ = 17.2 ± 0.3%) and normoxic (FiO_2_ = 20.9%) conditions, and both groups performing identical 12-week training protocols. Participants kept their habitual diet and physical activity, and evaluations showed no significant changes before and after the intervention. These findings support the inference that the observed fat loss was primarily attributable to the hypoxic stimulus. Consistently, a recent pilot study investigated the effects of different interventions in women with first-degree obesity, including normoxic training (exercise at 200 m altitude), IHT (exercise at simulated 2500 m, FiO_2_ = 15.4%), and IHE (passively expose at simulated 2500 m, FiO_2_ = 15.4%), focusing on BMI reduction and aerobic capacity improvement (Bagińska et al., 2024). The findings demonstrated that the IHT regime resulted in superior outcomes, substantially reducing body fat and body mass and enhancing aerobic capacity. However, it is worth noting that this was a small-sample, short-term pilot study with several methodological limitations. Specifically, the study did not control dietary intake, monitor physical activity, analyze blood biochemistry, or perform follow-up assessments. These preliminary results reflect that hypoxia-based interventions have a role in metabolic regulation in overweight or obese women, but this requires confirmation through more rigorous experimental designs.

**Table 2:** Clinical evidence of IHC in obesity and its comorbidities^*^.

No.	Subject	Age (years)	Sample size	IHC modality	Exercise protocol	Hypoxic protocol	Equipment	Outcomes	Adverse event	Mechanism	Ref.
1	Overweight/obese women	41.15 ± 8.88	82	IHT	12 weeks of cycle ergometer training (3 sessions/week)	FiO_2_ = 17.20 ± 0.30%	Normobaric hypoxia chamber	Reduce body fat content, increase muscle mass	NO	Not investigated	(Alba et al., 2018c)
2	Overweight/obese women	40.89 ± 8.28	82	IHT	12 weeks of cycle ergometer training (3 sessions/week)	FiO_2_ = 17.2 0± 0.30%	Normobaric hypoxia chamber	Improve cardiometabolic risk markers	NO	Not investigated	(Alba et al., 2018b)
3	Overweight/obese women	40.60 ± 9.50	59	IHT	12 weeks of cycle ergometer training (3 sessions/week)	FiO_2_ = 17.20 ± 0.30%	Normobaric hypoxia chamber	Reduce fat mass in the trunk	NR	Not investigated	(Alba et al., 2019)
4	Women with first-degree obesity	41.30 ± 10.50	41	IHE, IHT	4 weeks of cycle ergometer training (3 times/week)	FiO_2_ = 15.40%	Hypoxic thermoclimatic chamber	Reduce body fat and body mass, increase aerobic capacity	NO	Not investigated	(Bagińska et al., 2024)
5	Overweight/obese adults	36.03 ± 10.48	37	IHT	4-week medium-to-high intensity exercise (60 min, 5 times/week)	FiO_2_ = 15.00%	Normobaric hypoxic room	Reduce visceral fat mass, improve vascular elasticity	NR	Activation of SIRT1/HIF-1α/VEGF and SIRT1/PPARγ pathways	(Jiao et al., 2024)
6	Obese young adults	17-25	22	IHT	4 weeks, 8 aerobic sessions (running, stepping, cycling, dumbbell) + 3 strength sessions/week	FiO_2_ = 14.50-16.40%	Hypoxic training room	Reduce body weight, improve systolic blood pressure and mean blood pressure	NR	Not investigated	(Zhaowei et al., 2013)
7	Overweight/obese adults	51.00 ± 8.30	31	IHT	8 weeks of cycle ergometer training (24 sessions, 3 sessions/week)	FiO_2_ = 12.00%	Facemask breathing via a gas-mixing device	Improve exercise capacities, but no significant intergroup differences	NR	Not investigated	(Abdallah et al., 2022)
8	Obesity women with pre-diabetes	49	1	IHE	No exercise	Hypoxia (6 min, SpO2 83.00-91.00%)/normoxia (3 min) cycles, 1 h/day for 4 weeks	Altitude training device	Reduce body weight, decrease fasting blood glucose#	NR	Not investigated	(Nicholas and Rosalba, 2016)
9	Obese men with SAHS	25-50	49	IHT	13 weeks, 2 sessions/week, 1 h/session (15 min cycling + 15 min lifting 4-kg weights)	FiO_2_ = 16.00% for the first 2 weeks, then 13.70-14.80% for the remaining weeks	Hypoxia generator	Reduce body weight, BMI, and waist circumference, reduce energy intake, protect cardiovascular health	NR	Not investigated	(P et al., 2015)
10	Obese men with metabolic syndrome	Average 57	21	IHT	6 weeks of moderate-intensity treadmill exercise (60 min, 3 times/week)	FiO_2_ = 15.00%	Normobaric hypoxia chamber	Increase insulin sensitivity	NR	Activation of HIF-1α/VEGF pathway	(Knut et al., 2019)

^*^ IHC, intermittent hypoxic conditioning; IHE, intermittent hypoxic exposure; IHT, intermittent hypoxic training; FiO_2_, fraction of inspired oxygen; SpO_2_, blood oxygen concentrations; NAFLD, non-alcoholic fatty liver disease; SAHS, sleep apnea-hypopnea syndrome; NO indicates that the original study stated that no adverse events occurred; NR indicates that the original study did not report whether any adverse events occurred.

^#^ This is a single case report with concurrent diet modification; therefore, the observed effects cannot be solely attributed to IHE.

Beyond female populations, similar findings have also been observed in mixed-sex cohorts. Jiao et al. reported that under well-controlled caloric intake, overweight or obese adults (both males and females) who completed a 4-week IHT program (FiO_2_ = 15%, moderate-to-high-intensity exercise) exhibited clinically relevant reductions in BMI and visceral fat mass, along with favorable changes in vascular elasticity, compared with those undergoing normoxic training alone (Jiao et al., 2024).

As obesity prevalence among young adults has risen rapidly over the last decades, definitive evidence is critical to guide effective treatment strategies for these populations (Xiong-Fei et al., [[NoYear]]; Cole et al., 2020). Kong et al. conducted a 4-week weight loss residential training camp under military-style management in obese young adults following either IHT or normoxic training (Zhaowei et al., 2013). During the intervention, accommodation, diet, and training were uniformly controlled, and daily caloric deficits were comparable between groups. Under these strictly controlled conditions, participants in the IHT group lost more weight than those in the normoxic training group. Moreover, the IHT group exhibited measurable improvements in systolic blood pressure and mean blood pressure. This cardiometabolic benefit is likely attributed to the hypoxia-induced increase in arteriole diameter, which promotes peripheral vasodilation and lowers blood pressure (Aritz et al., 2011).

Overall, evidence from systematic reviews and meta-analyses suggests that intermittent hypoxia combined with exercise therapy can influence certain outcomes in obese adults, including body weight, BMI, waist circumference, waist-to-hip ratio, and fat mass, along with favorable changes in lean mass and cardiometabolic parameters (Alba et al., 2018a; Domingo et al., 2019). However, the current findings remain mixed and appear protocol-dependent. Indeed, some findings suggest that IHC does not offer superior benefits compared to normoxic exercise for certain outcome measures. For instance, a prospective, randomized controlled, single-blind study in overweight/obesity subjects observed that while an 8-week high-intensity intermittent training substantially improved exercise improved exercise capacities under both normobaric hypoxia and normoxia conditions, no significant intergroup differences were observed (Abdallah et al., 2022). In addition, the study found no meaningful improvements in vascular function, blood metabolism, or body adiposity parameters in either group (Abdallah et al., 2022). This study included 31 participants (16 in the hypoxic group, 15 in the normoxic group) with a mean age of approximately 50 years (23 males, 8 females), and its conclusions could be influenced by the relatively small sample size as well as the specific age-sex distribution of the cohort. Notably, the hypoxic stimulus in this study was relatively severe (FiO_2_ ~12%). The combination of high-intensity exercise and such severe hypoxia probably imposed excessive physiological stress, which likely offset the possible additive benefits of hypoxic exposure. Moreover, as the researchers acknowledged, the lack of monitoring of habitual physical activity levels and dietary intake means that these factors were not necessarily balanced between groups, potentially influencing the results (Abdallah et al., 2022). Notably, although hypoxic training did not show statistical superiority, it enabled comparable exercise capacity improvements with a lower absolute workload, offering a potential advantage for obese individuals with frequent joint pain. Overall, these different outcomes highlight the necessity of conducting high-quality clinical investigations with standardized protocols, larger sample sizes, and rigorous control of potential confounders to further validate the efficacy and efficiency of IHT in obese populations.

### 5.2 Role of IHC in obesity with comorbidities

Whether IHC can ameliorate obesity-related comorbidities has also been investigated. The following sections summarize the current evidence.

For non-alcoholic fatty liver disease (NAFLD) and pre-diabetes, existing evidence focuses predominantly on IHE. Consistent with the previous reports that intermittent hypoxia can prevent steatosis in liver cells, the latest findings elucidate the possible applications of intermittent hypoxia in obesity-associated fatty liver disease (Ling et al., 2008; Chen et al., 2023). As shown in **Table 2**, in a murine model of NAFLD, IHE (2 min cycle: 20 s FiO_2_ 8%/100 s FiO_2_ 20.9%; 12 h/day) not only attenuated liver steatosis, but also modulated bile acids composition, producing a protective effect in obesity-induced NAFLD (Chen et al., 2023). However, it should be noted that these findings are based on preclinical research, and therefore direct extrapolation to clinical efficacy in humans with obesity-associated NAFLD requires further investigation. A case report described an obese patient diagnosed with pre-diabetes who achieved favorable outcomes in weight loss and glycemic control after diet modification plus IHE (4 weeks, 1 h/day, alternating 6 min hypoxia with SpO_2_ maintained at 83-91% and 3 min normoxia) (Nicholas and Rosalba, 2016). Although this case report offers basic evidence supporting the potential of IHE, the concurrent dietary modification during the intervention period prevents the observed outcomes from being attributed solely to IHE.

For sleep apnea-hypopnea syndrome (SAHS) and metabolic syndrome, existing evidence comes predominantly from IHT. It has been suggested that obese patients with SAHS may benefit from IHT, as exercise promotes peripheral circulation and oxygen utilization, and hypoxic training reduces body weight and improves the respiratory system, all of which improve SAHS. However, this proposal requires conceptual caution. As a highly prevalent sleep-related breathing disorder, SAHS is fundamentally a pathological form of intermittent hypoxia (Serebrovskaya et al., 2008). In SAHS, repeated apneic episodes during sleep result in brief but intense hypoxia and hypercapnia, promoting oxidative stress, systemic inflammation, and sympathetic overactivation (Lv et al., 2023). This pattern of hypoxic stimulus is inherently uncontrolled, which can lead to adverse consequences over time. In contrast, therapeutic IHC involves controlled, dose-defined, and monitored hypoxic exposure applied during wakefulness for limited durations, which triggers adaptive responses without inducing the deleterious effects associated with chronic uncontrolled hypoxia. Moreover, modalities such as IHT incorporate exercise, which independently improves cardiovascular and metabolic function (Qiu et al., 2023). The distinct hypoxia paradigms of SAHS and IHC produce divergent physiological effects. As reported in the literature, obstructive sleep apnea-associated hypoxia drives adverse physiological cascades that promote persistent hypertension and other comorbidities, whereas therapeutic IHT minimally activates or even attenuates these responses, illustrating how the nature of the hypoxic stimulus determines whether the outcome is pathogenic or potentially beneficial (Serebrovskaya et al., 2008). Supporting this premise, a study involving obese males with SAHS concluded that under conditions where both groups followed a healthy dietary pattern, IHT not only effectively reduced body weight, BMI, and waist circumference, but also showed specific benefits in terms of reducing energy intake and protecting cardiovascular health (P et al., 2015). Notably, obesity is a major cause of respiratory compromise, characterized by reduced static and dynamic pulmonary volumes (Shah and Kaltsakas, 2023). The coexistence of obesity with SAHS may further exacerbate pulmonary dysfunction and impairs alveolar-capillary diffusion (Rouatbi et al., 2020). Therefore, safety monitoring, particularly careful SpO_2_ and symptom monitoring, is especially important when applying hypoxic exercise in obese patients with SAHS. In general, obese patients are at high risk of developing metabolic syndrome (Engin, 2024). In a randomized controlled trial, it was observed that a 6-week IHT (FiO_2_ = 15%) favorably affected insulin sensitivity in obese men with metabolic syndrome, whereas normoxic exercise (FiO_2_ = 21%) did not produce a comparable effect (Knut et al., 2019). However, although the two groups were well-matched at baseline in key characteristics (e.g., age, BMI, resting energy expenditure, blood lipid profiles) and peak exercise workload, dietary intake was not strictly controlled during the intervention. Thus, the potential confounding effect of unsupervised diet cannot be excluded when attributing the improvement in insulin sensitivity solely to IHT. Together, these initial findings reveal the applicability of both IHE and IHT in obese patients with comorbidities, providing preliminary clinical evidence for the application of IHC as an adjunctive management strategy in this population.

### 5.3 Determinants of the IHC response in obesity

As summarized above, the effects of IHC in obese individuals range from clear benefits to null outcomes. These controversial findings stem from several factors, including IHC protocols (e.g., hypoxic dose, exercise intensity and modality, session frequency, intervention duration), population characteristics (e.g., age, sex, baseline metabolic status), sample size, outcome measures, and confounder control (e.g., dietary intake, physical activity, sleep, and medication use). Among these, the hypoxic dose, the exercise protocol, and participant characteristics are discussed in detail below, as they directly modulate the physiological stimulus of IHC or influence how individuals respond to a given stimulus.

The first is the hypoxic dose, which encompasses FiO_2_, exposure duration, and cycle frequency. The magnitude of hypoxia can be described by FiO_2_ thresholds and their corresponding altitude equivalents, ranging from mild (FiO_2_ 0.16-0.18; 500–2000 m) to moderate (FiO_2_ 0.15-0.16; 2000–3000 m), severe (FiO_2_ 0.12-0.14; 3000–5000 m), and very severe (FiO_2_ < 0.12; > 5000 m) (Forbes et al., 2026). Controlled intermittent hypoxia can trigger adaptive physiological responses. For instance, mild hypoxia enhances ventilation, upper airway muscle activity, and limb motor recovery, which constitute the physiological basis for the health benefits of IHC (Puri et al., 2021). Consistent with this, studies reporting favorable outcomes in obese individuals have typically employed mild to moderate FiO_2_ levels (e.g., 15-17%) with normoxic recovery between cycles (Zhaowei et al., 2013; P et al., 2015; Nicholas and Rosalba, 2016; Alba et al., 2018c; Alba et al., 2018b; Alba et al., 2019; Knut et al., 2019; Bagińska et al., 2024; Jiao et al., 2024). In contrast, the trial that found no superiority of IHC used a more severe stimulus (FiO_2_ ~12%) (Abdallah et al., 2022). Available evidence indicates that excessive or prolonged hypoxia may override adaptive mechanisms and trigger oxidative stress, systemic inflammation, and sympathetic overactivation, ultimately resulting in tissue injury, which may help explain this null finding (Dantu et al., 2025). Thus, the benefits of IHC appear to be confined to an optimal range of hypoxic dose, where insufficient hypoxia fails to induce adaptation and excessive hypoxia produces deleterious effects.

The second factor is the exercise protocol. The type, intensity, frequency, and duration of exercise varied considerably across studies, ranging from cycling and treadmill running to combined aerobic and resistance training (Zhaowei et al., 2013; P et al., 2015; Alba et al., 2018c; Alba et al., 2018b; Alba et al., 2019; Knut et al., 2019; Abdallah et al., 2022; Bagińska et al., 2024). These differences in exercise modality may independently influence intervention outcomes, adding another layer of variability to cross-study comparisons. Moreover, because hypoxia increases the physiological burden of exercise through reduced oxygen availability and heightened ventilatory demand, the same absolute workload elicits a higher relative intensity under hypoxia (Forbes et al., 2026). This elevation in relative intensity may partly account for the superior metabolic effects of IHC over normoxic training at matched absolute workloads. However, when exercise intensity is already high, superimposing hypoxia may push physiological stress beyond the adaptive range, thereby increasing the risk of adverse responses such as excessive desaturation or cardiovascular strain (Xu et al., 2021; Forbes et al., 2026). Thus, the net effect of combining hypoxia with exercise depends critically on the relative dose of each stimulus.

The third factor is participant characteristics, which differed considerably among the included trials. Even under identical hypoxic and exercise protocols, the physiological burden experienced by different individuals can vary substantially. For example, obese individuals with respiratory compromise, which is more prevalent among those with severe obesity, may experience a greater physiological burden under hypoxia, manifesting as more pronounced desaturation, exaggerated hemodynamic responses, or reduced exercise tolerance (Krishnan et al., 2006; Akshay, 2009; Vultur et al., 2025). This may partly explain why IHC produces adverse rather than beneficial outcomes in certain obese subgroups. Sex distribution also varied across studies, yet direct comparisons of responses between males and females are currently lacking (Alba et al., 2018c; Alba et al., 2018b; Alba et al., 2019; Jiao et al., 2024). In addition, the baseline exercise capacity of participants has not been systematically documented and may influence the metabolic response to IHC. Thus, the outcome of IHC depends not only on the hypoxic dose and exercise protocol, but also on the characteristics of the population studied.

Besides, the duration of intervention also varied across studies, and the optimal treatment length for inducing metabolic benefits has not been established, representing an additional source of heterogeneity when comparing outcomes across trials (Zhaowei et al., 2013; P et al., 2015; Nicholas and Rosalba, 2016; Alba et al., 2018c; Alba et al., 2018b; Alba et al., 2019; Knut et al., 2019; Bagińska et al., 2024; Jiao et al., 2024). Together, the above factors influence whether IHC ultimately leads to an adaptive or maladaptive response, providing a mechanistic explanation for the inconsistent findings in the literature.

### 5.4 Mechanisms of IHC in combating obesity and its complications

**Figure 4:**
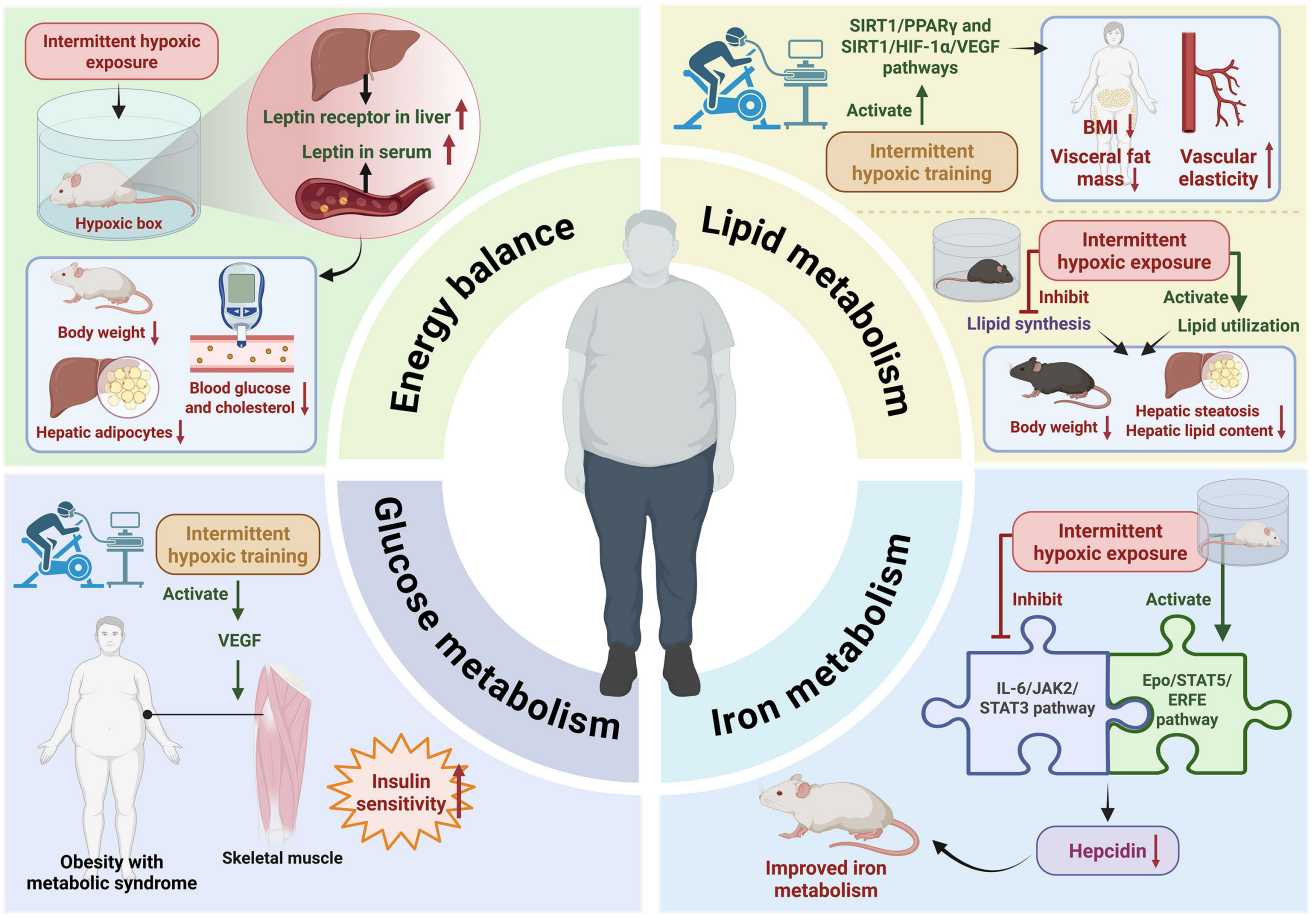
The proposed mechanisms by which IHC ameliorates obesity and its related complications.

To understand how IHC may exert its effects in obesity, it is essential to first discuss the key pathophysiological features of obesity. Under normal conditions, the human body can flexibly switch between carbohydrate and fat oxidation in response to changes in energy supply and demand, a capacity termed metabolic flexibility (Zhang et al., 2025). This process relies heavily on mitochondrial function, as mitochondrial adaptation to environmental changes may represent a core component of metabolic flexibility (Goodpaster and Sparks, 2017; Tsilingiris et al., 2021). In obesity, metabolic flexibility is impaired, resulting in disrupted energy homeostasis and metabolic dysfunction (Kong et al., 2025). This impairment is particularly evident in skeletal muscle and adipose tissue, which play central roles in maintaining whole-body metabolic flexibility (Goodpaster and Sparks, 2017). In skeletal muscle, obesity compromises mitochondrial biogenesis and function, shifting the metabolic fate of fatty acids from oxidation toward storage and thereby promoting intramyocellular lipid accumulation and insulin resistance (Jun et al., 2024). In adipose tissue, obesity drives adipocyte hypertrophy and local hypoxia, which promote immune cell infiltration and pro-inflammatory cytokine secretion. These events disrupt normal adipose microenvironment and lipid handling, elevating levels of circulating free fatty acids, which in turn contribute to ectopic lipid deposition in the liver and skeletal muscle and insulin resistance (Kawai et al., 2021). These processes are interconnected in obesity, forming a self-reinforcing pathological network that perpetuates systemic metabolic dysfunction. How IHC may modulate these complex pathological processes in obesity and its complications remains incompletely understood, and the available evidence is discussed below (**Figure 4**; **Supplementary Figure 1**).

#### 5.4.1 Energy balance

Leptin, a 16 kDa hormone encoded by the ob/ob gene and predominantly secreted by adipose tissue, is an important substance involved in the regulation of food intake, body weight, and reproductive function (Obradovic et al., 2021; Fan et al., 2023). It balances energy metabolism by acting on leptin receptors and thereby activating anorexigenic neuronal pathways (Heike, 2009; Seth et al., 2020). Numerous studies have indicated that leptin or leptin receptor signaling deficiency plays a central role in obesity development, which has made leptin a key target for obesity intervention research (Genchi et al., 2021). Ling et al. found that 40-day IHE significantly reduced body weight, blood glucose, and blood cholesterol, and effectively decreased the distribution density and scope of hepatic adipocytes in obese female mice (Ling et al., 2008). Combined with the observed IHE-induced elevation in serum leptin concentration and hepatic leptin receptor expression, the researchers hypothesized that the regulatory effects of IHC on body nutritional metabolism may involve the leptin/leptin receptor signaling pathway (Ling et al., 2008). However, these observations are derived from animal evidence. Reliable experimental validation of this hypothesis in humans is lacking, and it remains unknown whether there is a causal relationship between leptin signaling modulation and IHC-mediated metabolic improvements in obese patients. Notably, the interpretation of these findings requires caution in light of leptin resistance, a condition commonly present in obesity in which circulating leptin levels are elevated but central leptin signaling is impaired (Obradovic et al., 2021). In this context, the IHE-induced elevation in serum leptin and hepatic leptin receptor expression may reflect compensatory upregulation rather than restoration of leptin sensitivity, and whether these molecular changes are causally linked to the metabolic benefits of IHC remains uncertain. Future studies should therefore assess the effects of IHC on functional leptin sensitivity rather than relying solely on expression data.

#### 5.4.2 Lipid metabolism

SIRT1, a deacetylase belonging to the sirtuin group, plays a crucial regulatory role in maintaining lipid metabolism homeostasis (Bao-Fei et al., 2023; Shan et al., 2023). Furthermore, SIRT1 activation represents an emerging treatment target for obesity and its associated complications (Abduraman et al., 2021; Quiñones et al., 2021). Mechanistically, SIRT1 suppresses peroxisome proliferator-activated receptor-γ (PPARγ), a key regulator of adipogenesis and lipid metabolism, thereby inhibiting fat accumulation and promoting lipolysis (Jiali et al., 2022). Additionally, SIRT1 can directly bind to and deacetylate hypoxia-inducible factor-1α (HIF-1α), consequently upregulating the expression of HIF-1α target gene vascular endothelial growth factor (VEGF), which facilitates vasodilation and angiogenesis (Hyun-Yoo et al., 2015). Data obtained by Jiao et al. confirmed that overweight or obese adults in the IHT group showed significant reductions in BMI and visceral fat mass, along with favorable changes in vascular elasticity, compared with normoxic training alone (Jiao et al., 2024). Further investigation suggested that the regulatory effects of IHT on lipid metabolism and vascular homeostasis may be associated with the activation of SIRT1/PPARγ and SIRT1/HIF-1α/VEGF pathways. Among the downstream mediators of these pathways, HIF-1α is of particular interest because its activation in adipose tissue has been reported to produce opposing metabolic effects (Ban et al., 2014; Gonzalez et al., 2018). In the obese state, adipose tissue hypoxia leads to accumulation of HIF-1α in adipocytes (Gonzalez et al., 2018). Some studies indicate that it protects against obesity and insulin resistance by stimulating thermogenic functions of brown adipose tissue, whereas others demonstrate that it exacerbates adipose tissue inflammation, fibrosis, and insulin resistance (Ban et al., 2014; Gonzalez et al., 2018). In light of these divergent findings, whether IHC-mediated upregulation of HIF-1α in obese individuals exerts beneficial or detrimental metabolic effects cannot be determined from current evidence, and further studies are needed to clarify the net consequences of modulating this pathway.

NAFLD, the most prevalent chronic liver disorder worldwide, represents the hallmark hepatic manifestation of obesity-associated metabolic dysfunction (Cotter and Rinella, 2020; Piester et al., 2023). The current view is that the treatment of NAFLD is primarily focused on treating obesity. The therapeutic potential of IHC has attracted attention to this obesity-associated complication. In a murine model of NAFLD, IHE notably reduced body weight, alleviated hepatic steatosis, and decreased hepatic lipid content (Chen et al., 2023). These lipid metabolic improvements were attributed to enhanced lipid utilization and inhibition of lipid synthesis by regulating typical enzymes or transcriptional factors (Chen et al., 2023). The underlying mechanisms primarily consisted of the up-regulation of lipolytic enzymes, including phosphorylated hormone-sensitive lipase (p-HSL), adipose triglyceride lipase (ATGL), and the rate-limiting enzyme for fatty acid β-oxidation, carnitine palmitoyl transferase-1α (CPT-1α). Concurrently, IHE down-regulated the regulators of lipid synthesis and fatty acid uptake, including PPARγ, fatty acid transport protein 5 (Fatp5), carbohydrate response element-binding protein (Chrebp), sterol regulatory element-binding protein-1c (Srebp-1c), and stearoyl-CoA desaturase-1 (Scd-1), which ultimately attenuated hepatic lipid accumulation and prevented progression of liver disease.

#### 5.4.3 Iron metabolism

There is growing evidence that dysregulated iron homeostasis frequently occurs in obese individuals and may progress to obesity-related iron deficiency and ultimately anemia (González-Domínguez et al., 2020; Qiu et al., 2022). Notably, Cui et al. observed that IHE was effective in alleviating multiple obesity-related pathological manifestations in diet-induced obese rats, with particular efficacy in normalizing iron metabolism (Cui et al., 2020). It was further found that this improvement was attributed to the down-regulation of hepcidin by decreasing interleukin6 (IL-6) and increasing erythropoietin (Epo) (Cui et al., 2020). These findings were subsequently validated in a metabolic syndrome model, where improvement in iron metabolism disorders induced by chronic intermittent hypobaric hypoxia was associated with inhibition of IL-6/Janus kinase 2 (JAK2)/signal transducer and activation of the transcription 3 (STAT3) and activation of the Epo/STAT5/erythroferrone (ERFE) signaling pathway (Cui et al., 2023). Together, these studies offer emerging evidence that hypoxia-based therapies can effectively target IL-6 and Epo to restore iron balance in obesity-related metabolic disorders.

#### 5.4.4 Glucose metabolism

Preliminary findings indicate that IHC plays a beneficial role in glucose metabolism, with insulin resistance being a key target of its action. Notably, the metabolic benefits of IHC appear to be highly dependent on the hypoxic dose. A recent study by Wang et al. demonstrated that during an acute bout of low-intensity exercise, moderate hypoxia (FiO_2_ ~16%) provided a more favorable balance for glucose regulation compared with more severe hypoxic exposure (FiO_2_ ~14.8%) in overweight adults, supporting the notion that the hypoxic dose should be carefully optimized when targeting metabolic benefits (Tee et al., 2023b). Insulin resistance, defined as a reduced effect of insulin on glucose metabolism at a given insulin concentration, has become increasingly prevalent largely driven by rising obesity levels. This condition impairs normal glucose homeostasis and in turn promotes obesity-related cardiometabolic complications such as metabolic syndrome (Rocco et al., 2018). Previous studies have elucidated that hypoxic exercise enhances glucose tolerance by increasing peripheral insulin sensitivity, thereby establishing a link between hypoxic training and improved insulin resistance (Richard et al., 2011). In a randomized controlled trial, skeletal muscle insulin sensitivity was significantly increased after IHT in middle-aged obese men with metabolic syndrome (Knut et al., 2019). This study also observed that hypoxic training resulted in increased plasma levels of VEGF. Notably, VEGF is regulated by HIF-1α, an important metabolic regulator that contributes to insulin sensitivity by promoting angiogenesis in adipose tissue (Fujisaka, 2021; Hai et al., 2024). However, whether activation of the HIF-1α/VEGF pathway is causally responsible for the improved insulin sensitivity observed after IHT has not been directly demonstrated. In addition, the combination of hypoxia (14% O_2_, 12 h/day) and exercise training (swimming, 3 h twice daily) was associated with reduced body mass, improved glucose tolerance, and increased glucose transporter 4 (GLUT4) protein expression in skeletal muscle of rats (Li-Ling et al., 2004). It is well known that insulin mediates muscle glucose uptake by translocating the GLUT4 to the cell surface, with impaired GLUT4 translocation being a key feature of insulin resistance (van Gerwen et al., 2023; Hu et al., 2025). However, direct evidence that GLUT4 upregulation is responsible for the effects of hypoxic intervention on insulin resistance is still lacking, and the functional significance of these changes for hypoxic intervention-induced metabolic improvements awaits further investigation.

## 6 Safety of IHC in obesity

As research on the therapeutic potential of IHC in obesity grows, its safety profile in this population warrants equal attention. Available studies suggest that IHC is generally well-tolerated in selected participants, but systematic safety data in obese populations remain limited (Alba et al., 2018c; Alba et al., 2018b; Bagińska et al., 2024). It is crucial to recognize that hypoxic interventions represent a double-edged sword for obese individuals, and whether the outcome is beneficial or harmful depends on multiple factors (T et al., 2016).

As discussed in Section 3.1, obesity impairs respiratory function through mechanical constraints, heightened ventilatory demand, and reduced respiratory efficiency, all of which increase the risk of rapid desaturation during hypoxic exposure (Krishnan et al., 2006; Akshay, 2009; Michael and Hans-Joachim, 2012). As reported by Xu et al., an obese individual with a baseline SpO_2_ of 96% developed severe hypoxemia (SpO_2_ = 72%) and tachycardia (heart rate = 97 bpm) during acute hypoxic exposure. The protocol consisted of 6 h of acute hypoxia (FiO_2_ = 11.7-11.2%), 30 min of hypoxic rest, followed by 30 min of hypoxic exercise, and then 5 h of hypoxic rest. Acute hypoxic exercise further aggravated hypoxemia, with SpO_2_ dropping to 59%, and induced marked autonomic dysfunction and subsequently severe acute mountain sickness (Xu et al., 2021). This case illustrates that combined severe hypoxia and exercise poses a serious risk in obese individuals, highlighting the need for appropriate IHC protocol design and close safety monitoring.

In addition, intermittent hypoxia exerts dose-dependent effects on the cardiovascular system (Dantu et al., 2025). Specifically, mild and controlled hypoxic exposure appears to induce adaptive responses (e.g., enhanced angiogenesis and improved oxygen utilization), whereas severe or prolonged hypoxia can promote cardiovascular pathology via oxidative stress, endothelial dysfunction, and sympathetic overactivation (Dantu et al., 2025). Obesity also contributes to cardiovascular damage through chronic low-grade inflammation, heightened oxidative stress, and sympathetic overactivation (Isabel et al., 2012; Omair and Travis, 2020; Figorilli et al., 2025). These pre-existing conditions may lower the threshold for hypoxia-induced cardiovascular complications. Therefore, in obese patients, uncontrolled hypoxic exposure may amplify these underlying risks, increasing the likelihood of adverse cardiovascular events.

Importantly, safety considerations for IHC differ by obesity phenotype, as the underlying pathophysiology and comorbidity burden vary substantially across subgroups. Obese individuals complicated by SAHS require specific attention, as the pre-existing pathological intermittent hypoxia of SAHS may compound the effects of therapeutic hypoxic exposure, potentially amplifying oxidative stress and sympathetic overactivation (Lv et al., 2023). Similarly, while preliminary evidence suggests that IHC has favorable effects on vascular function in obese individuals, whether it represents a favorable risk-benefit option for those with obesity-related cardiovascular disease remains unclear (Zhaowei et al., 2013; Alba et al., 2018b; Jiao et al., 2024). These patients, who have adverse cardiac remodeling and compromised cardiac function, may therefore have a narrower therapeutic window for safe IHC application (Szabo et al., 2023). Dedicated studies with careful cardiovascular monitoring are warranted. Individuals with severe obesity (BMI ≥ 40 kg/m^2^) experience excessive fat accumulation in the chest and abdominal regions, which causes external compression of the airways and markedly impairs respiratory function, raising the susceptibility to obesity hypoventilation syndrome (OHS) (Vultur et al., 2025). OHS manifests as daytime hypercapnia, diminished central respiratory drive, and mechanical ventilatory constraints. Moreover, recurrent hypoxic episodes in OHS lead to oxidative stress and systemic inflammation, promoting hypertension and potentially progressing to heart failure (Vultur et al., 2025). Taking these factors into account, the possibility of increased risk of adverse respiratory and cardiovascular events during IHC in this population cannot be excluded. Thus, screening for OHS should be considered before initiating IHC in this subgroup, and lower exercise intensities, milder hypoxic doses, and close SpO_2_ monitoring are advisable. Sarcopenic obesity, characterized by low skeletal muscle mass and excess adiposity, has received increasing attention (Axelrod et al., 2023). Resistance and aerobic training have been recommended for managing this condition (Borba and Costa, 2025). However, direct evidence on IHC in sarcopenic obesity is currently lacking. Prolonged intermittent hypoxia, as observed in conditions such as chronic obstructive pulmonary disease (COPD), has been shown to dysregulate skeletal muscle protein homeostasis and contribute to a sarcopenic phenotype (Attaway et al., 2023). This raises theoretical concerns that uncontrolled or excessive hypoxic exposure could negatively affect muscle mass in individuals with compromised muscle function. Therefore, if IHC were to be applied in this population, an appropriate IHC protocol should be carefully designed, along with close monitoring of muscle function and body composition.

Among the IHC studies in obese populations included in this review, only three explicitly stated that no adverse events were recorded during the intervention. The remaining studies did not report on adverse events (**Table 2**). This likely reflects insufficient monitoring rather than the true absence of risk, emphasizing the need for more rigorous safety surveillance. To ensure the safety of IHC in obese populations, we recommend a multi-step safety framework. First, participants should be carefully screened for cardiovascular, respiratory, and metabolic health to confirm adequate physical fitness. Second, IHC protocols (e.g., hypoxic dose, exposure duration, cycle frequency, exercise intensity, and modality) should be tailored to individual risk profiles, as informed by the phenotype-specific considerations discussed above. For instance, individuals with SAHS may require lower hypoxic doses and closer monitoring than obese individuals without comorbidities. During IHC sessions, individuals should undergo close monitoring of key physiological parameters, such as SpO_2_, heart rate, and blood pressure, along with clinical symptoms. Standardized adverse event reporting should be adopted to systematically document any adverse events (e.g., hypoxemia, excessive dyspnea, dizziness, syncope, cardiovascular events), as well as their severity, duration, and relationship to the intervention. Furthermore, dose-stopping criteria, based on objective measures such as significant SpO_2_ decline, intolerable symptoms, or abnormal cardiorespiratory responses, should be established to terminate the intervention when necessary, thereby minimizing potential risks. In addition, emergency equipment (e.g., supplemental oxygen and resuscitation devices) and trained personnel should be available during all IHC sessions. Beyond immediate safety measures, the long-term safety of IHC in obese populations also requires attention. The studies included in this review were predominantly short-term, and no follow-up data exist on potential late-onset adverse effects. It also remains unknown whether the effects of IHC are maintained, diminished, or even reversed upon prolonged or repeated intervention, particularly in individuals with obesity-related comorbidities. Therefore, extended follow-up studies are needed to evaluate the long-term efficacy and safety of IHC across different obesity phenotypes.

## 7 Limitations and perspectives

Although the emerging role of IHC in obesity management has been suggested by several studies, there are still considerable limitations in the existing research (Zhaowei et al., 2013; Alba et al., 2018c; Alba et al., 2018b; Alba et al., 2019; Bagińska et al., 2024; Jiao et al., 2024). First, IHC regimens vary widely across studies in exercise parameters (e.g., mode, intensity, duration, frequency), hypoxic parameters (e.g., FiO_2_, altitude, SpO_2_ response, cycles, total period), as well as comparator type, dietary control, and intervention length. However, comparative research between these different IHC regimens remains scarce. Consequently, standardized protocols for determining the optimal IHC regimen for obese patients are still lacking. Second, there remains insufficient evidence regarding the applicability of IHC in different obesity subtypes and its long-term efficacy. Most included studies are short-term, and no follow-up data exist on the maintenance of weight loss, visceral fat reduction, or cardiometabolic improvements. Therefore, whether the beneficial effects of IHC can be sustained beyond the intervention period remains unclear, highlighting the need for future trials with extended follow−up assessments. Third, excessive or prolonged IHC exposure has the potential to increase the risk of cardiovascular and respiratory dysfunction, so the safe clinical application of IHC for obesity treatment requires careful consideration (Xu et al., 2021; Bae et al., 2025). Fourth, emerging studies have only identified preliminary anti-obesity mechanisms of IHC, and its more in-depth mechanism requires further investigation to provide an evidence-based basis for the widespread application of IHC (Ling et al., 2008; Cui et al., 2020; Jiao et al., 2024). In summary, we propose that future research in this field should focus on conducting large-scale, multicenter clinical trials that include matched normoxic comparator groups, exercise mode and workload-matched exercise, and rigorous dietary control. Additionally, standardized adverse-event reporting and follow-up assessments after intervention cessation are needed to evaluate long-term durability. These efforts will help clarify the efficacy of IHC in different obese populations and to identify the optimal IHC protocol to achieve maximum efficacy. Moreover, we believe that future research in this area should elucidate the precise mechanisms through which IHC exerts its anti-obesity effects.

## 8 Conclusion

IHC represents an emerging interventional approach under investigation for obesity management. This review summarized available evidence on the anti-obesity efficacy of IHC and explored its underlying mechanisms. The evidence accumulated in the review demonstrates that IHC can influence certain outcomes (e.g., body weight, visceral fat, and cardiometabolic parameters) in specific populations under some protocols. These characteristics suggest that IHC is being investigated as a possible adjunct to conventional obesity treatment. Although exercise in moderate hypoxia has shown preliminary effects, the available data remain heterogeneous and insufficient to support a universal recommendation for IHC in obesity treatment. Further research is needed to confirm its weight-loss efficacy and to establish standardized protocols. In addition, rigorous safety monitoring and evidence of long-term durability must be demonstrated before clinical implementation. Overall, we believe that IHC warrants further investigation as a supervised adjunctive strategy for selected individuals with obesity.

## References

[B1] Abdallah G. Samarmar C. Anna B. Lisa C. Stéphane D. Bernard W. . (2022). Hypoxic high-intensity interval training in individuals with overweight and obesity. Am. J. Physiol. Regul. Integr. Comp. Physiol. 323, R700–R709. doi: 10.1152/ajpregu.00049.2022 36121143

[B2] Abduraman A. Azizan N. Teoh S. Tan B. (2021). Ketogenesis and SIRT1 as a tool in managing obesity. Obes. Res. Clin. Pract. 15, 10–18. doi: 10.1016/j.orcp.2020.12.001 33371997

[B3] Afina B. Oleg G. Alexander P. Ines G. Alexander Yu K. Nikita V. . (2021). The effects of intermittent hypoxic–hyperoxic exposures on lipid profile and inflammation in patients with metabolic syndrome. Front. Cardiovasc. Med. 8, 700826. doi: 10.3389/fcvm.2021.700826 34513946 PMC8429814

[B4] Akshay S. (2009). Altered resting and exercise respiratory physiology in obesity. Clin. Chest Med. 30, 445–454. doi: 10.1016/j.ccm.2009.05.003 19700043 PMC2765111

[B5] Alba C.-C. Marta C.-C. Darrell C. Rafael T. Guillermo O. Javier B.-S. (2018a). Effects training in hypoxia on cardiometabolic parameters in obese people: A systematic review of randomized controlled trial. Aten Primaria 51, 397–405. doi: 10.1016/j.aprim.2018.03.011 30172575 PMC6837087

[B6] Alba C.-C. Marta C.-C. Guillermo O. Rafael T. Javier B.-S. (2019). Detraining effect on overweight/obese women after high-intensity interval training in hypoxia. Scand. J. Med. Sci. Sports 29, 535–543. doi: 10.1111/sms.13380 30615248

[B7] Alba C.-C. Marta C.-C. Javier B.-S. Martin B. Rafael T. Guillermo O. (2018b). Effects of high-intensity interval training under normobaric hypoxia on cardiometabolic risk markers in overweight/obese women. High Alt. Med. Biol. 19, 356–366. doi: 10.1089/ham.2018.0059 30204493

[B8] Alba C.-C. Marta C.-C. Martin B. Ismael M.-G. Rafael T. Javier B.-S. . (2018c). High-intensity interval training in normobaric hypoxia leads to greater body fat loss in overweight/obese women than high-intensity interval training in normoxia. Front. Physiol. 9, 60. doi: 10.3389/fphys.2018.00060 29472870 PMC5810257

[B9] Aritz U. Pedro G. María P. Alfredo J. (2011). Usefulness of combining intermittent hypoxia and physical exercise in the treatment of obesity. J. Physiol. Biochem. 68, 289–304. doi: 10.1007/s13105-011-0115-1 22045452

[B10] Attaway A. Bellar A. Mishra S. Karthikeyan M. Sekar J. Welch N. . (2023). Adaptive exhaustion during prolonged intermittent hypoxia causes dysregulated skeletal muscle protein homeostasis. J. Physiol. 601, 567–606. doi: 10.1113/jp283700 36533558 PMC10286804

[B11] Axelrod C. L. Dantas W. S. Kirwan J. P. (2023). Sarcopenic obesity: emerging mechanisms and therapeutic potential. Metabolism 146, 155639. doi: 10.1016/j.metabol.2023.155639 37380015 PMC11448314

[B12] Bae J. Nelson L. Boye K. Mather K. (2025). Prevalence of complications and comorbidities associated with obesity: a health insurance claims analysis. BMC Public Health 25, 273. doi: 10.1186/s12889-024-21061-z 39844122 PMC11756071

[B13] Bagińska M. Kałuża A. Tota Ł. Piotrowska A. Maciejczyk M. Mucha D. . (2024). The impact of intermittent hypoxic training on aerobic capacity and biometric-structural indicators among obese women—a pilot study. J. Clin. Med. 13, 380. doi: 10.3390/jcm13020380 38256514 PMC10816855

[B14] Ban J. J. Ruthenborg R. J. Cho K. W. Kim J. W. (2014). Regulation of obesity and insulin resistance by hypoxia-inducible factors. Hypoxia (Auckl) 2, 171–183. doi: 10.2147/hp.S68771 27774475 PMC5045065

[B15] Bao-Fei Y. Lan-Fen L. Yi-Fang Z. Qian L. Jing-Zheng Z. Yi-Feng X. . (2023). Huangqin decoction alleviates lipid metabolism disorders and insulin resistance in nonalcoholic fatty liver disease by triggering SIRT1/NF-κB pathway. World J. Gastroenterol. 29, 4744–4762. doi: 10.3748/wjg.v29.i31.4744 37664157 PMC10473922

[B16] Beshir A. Elnour A. Soorya A. Mohamed A. Goh S. Hussain H. . (2023). A narrative review of approved and emerging anti-obesity medications. Saudi Pharm. J. 31, 101757. doi: 10.1016/j.jsps.2023.101757 37712012 PMC10497995

[B17] Blüher M. (2019). Obesity: Global epidemiology and pathogenesis. Nat. Rev. Endocrinol. 15, 288–298. doi: 10.1038/s41574-019-0176-8 30814686

[B18] Borba V. Costa A. (2025). Sarcopenic obesity: a review. Arch. Endocrinol. Metab. 68, e240084. doi: 10.20945/2359-4292-2024-0084 40215288 PMC11967173

[B19] Bray G. A. (2025). Obesity: A 100 year perspective. Int. J. Obes. (Lond) 49, 159–167. doi: 10.1038/s41366-024-01530-6 38714830

[B20] Bray G. A. Ryan D. H. (2021). Evidence-based weight loss interventions: Individualized treatment options to maximize patient outcomes. Diabetes Obes. Metab. 23, 50–62. doi: 10.1111/dom.14200 32969147

[B21] Burtscher J. Citherlet T. Camacho-Cardenosa A. Camacho-Cardenosa M. Raberin A. Krumm B. . (2024). Mechanisms underlying the health benefits of intermittent hypoxia conditioning. J. Physiol. 602, 5757–5783. doi: 10.1113/jp285230 37860950

[B22] Busebee B. Ghusn W. Cifuentes L. Acosta A. (2023). Obesity: A review of pathophysiology and classification. Mayo Clin. Proc. 98, 1842–1857. doi: 10.1016/j.mayocp.2023.05.026 37831039 PMC10843116

[B23] Chakhtoura M. Haber R. Ghezzawi R. Rhayem C. Tcheroyan R. Mantzoros C. S. (2023). Pharmacotherapy of obesity: An update on the available medications and drugs under investigation. EClinicalMedicine 58, 101882. doi: 10.1016/j.eclinm.2023.10188 36992862 PMC10041469

[B24] Chandrasekaran P. Weiskirchen R. (2024). The role of obesity in type 2 diabetes mellitus—an overview. Int. J. Mol. Sci. 25, 1882. doi: 10.3390/ijms25031882 38339160 PMC10855901

[B25] Chen Y. Wang L. Zheng X. Zhang J. Sun H. Chen X. . (2023). Improvement of obesity-induced fatty liver disease by intermittent hypoxia exposure in a murine model. Front. Pharmacol. 14, 1097641. doi: 10.3389/fphar.2023.1097641 36873991 PMC9974667

[B26] Chia-Hua K. Brennan M. (2016). Abdominal fat reducing outcome of exercise training: Fat burning or hydrocarbon source redistribution? Can. J. Physiol. Pharmacol. 94, 695–698. doi: 10.1139/cjpp-2015-0425 27152424

[B27] Cole D. Aya B. Orit P.-H. Arnon A. Gilad T. (2020). Cardiovascular morbidity, diabetes and cancer risk among children and adolescents with severe obesity. Cardiovasc. Diabetol. 19, 79. doi: 10.1186/s12933-020-01052-1 32534575 PMC7293793

[B28] Consultation (2000). Obesity: Preventing and managing the global epidemic. World Health Organ. Tech. Rep. Ser. 894, 1–253. 11234459

[B29] Conway B. Rene A. (2004). Obesity as a disease: No lightweight matter. Obes. Rev. 5, 145–151. doi: 10.1111/j.1467-789X.2004.00144.x 15245383

[B30] Cotter T. G. Rinella M. (2020). Nonalcoholic fatty liver disease 2020: the state of the disease. Gastroenterology 158, 1851–1864. doi: 10.1053/j.gastro.2020.01.052 32061595

[B31] Courcoulas A. P. Daigle C. R. Arterburn D. E. (2023). Long term outcomes of metabolic/bariatric surgery in adults. BMJ 383, e071027. doi: 10.1136/bmj-2022-071027 38110235

[B32] Coutant R. Bouhours-Nouet N. Donzeau A. Fauchard M. Decrequy A. Malka J. . (2017). Bariatric surgery in adolescents with severe obesity: Review and state of the art in France. Ann. Endocrinol. (Paris) 78, 462–468. doi: 10.1016/j.ando.2017.03.002 28870706

[B33] Cui F. Guo Y. Hu S. Zhang J. Shi M. (2020). Chronic intermittent hypobaric hypoxia improves markers of iron metabolism in a model of dietary-induced obesity. J. Inflamm. (Lond) 17, 36. doi: 10.1186/s12950-020-00265-1 33292270 PMC7648949

[B34] Cui F. Sun H. Mi Y. Li Y. Tang Y. Wang H. . (2023). Chronic intermittent hypobaric hypoxia improves iron metabolism disorders via the IL-6/JAK2/STAT3 and EPO/STAT5/ERFE signaling pathways in metabolic syndrome rats. J. Trace Elem. Med. Biol. 79, 127259. doi: 10.1016/j.jtemb.2023.127259 37413927

[B35] Dantu G. Masetti S. Barsinge T. Madarapu A. Ragupathi S. Inoyatova M. . (2025). Cardiovascular implications of intermittent hypoxia: A comprehensive narrative review. Cureus 17, e97121. doi: 10.7759/cureus.97121 41426756 PMC12712449

[B36] Domingo J. Olivier C. Andrés P. Jacobo Á. (2019). Additive stress of normobaric hypoxic conditioning to improve body mass loss and cardiometabolic markers in individuals with overweight or obesity: A systematic review and meta-analysis. Physiol. Behav. 207, 28–40. doi: 10.1016/j.physbeh.2019.04.027 31047948

[B37] Eisenberg D. Shikora S. A. Aarts E. Aminian A. Angrisani L. Cohen R. V. . (2023). 2022 American Society of Metabolic and Bariatric Surgery (ASMBS) and International Federation for the Surgery of Obesity and Metabolic Disorders (IFSO) indications for metabolic and bariatric surgery. Surg. Obes. Relat. Dis. 19, 222–234. doi: 10.1016/j.soard.2022.08.013 36280539

[B38] Elena A. Natalia N. Mikhail Y. Ksenia A. (2022). Intermittent hypoxic training as an effective tool for increasing the adaptive potential, endurance and working capacity of the brain. Front. Neurosci. 16, 941740. doi: 10.3389/fnins.2022.941740 35801184 PMC9254677

[B39] Elmaleh-Sachs A. Schwartz J. L. Bramante C. T. Nicklas J. M. Gudzune K. A. Jay M. (2023). Obesity management in adults: A review. JAMA 330, 2000–2015. doi: 10.1001/jama.2023.19897 38015216 PMC11325826

[B40] Engin A. (2024). The definition and prevalence of obesity and metabolic syndrome: Correlative clinical evaluation based on phenotypes. Adv. Exp. Med. Biol. 1460, 1–25. doi: 10.1007/978-3-031-63657-8_1 39287847

[B41] Fan Y. Qin Y. Yuan W. Fan H. Lin H. (2023). Chemical shift assignments of wildtype human leptin. Biomol. NMR Assign 17, 265–268. doi: 10.1007/s12104-023-10153-3 37796383

[B42] Figorilli F. Velluzzi F. Redolfi S. (2025). Obesity and sleep disorders: a bidirectional relationship. Nutr. Metab. Cardiovasc. Dis. 35, 104014. doi: 10.1016/j.numecd.2025.104014 40180826

[B43] Fink J. Seifert G. Blüher M. Fichtner-Feigl S. Marjanovic G. (2022). Obesity surgery. Dtsch. Arztebl Int. 119, 70–80. doi: 10.3238/arztebl.m2021.0359 34819222 PMC9059860

[B44] Forbes S. Hearon C. Cillay J. Lawley J. Stembridge M. Subudhi A. . (2026). Exercise in acute and subacute hypoxic conditions: Physiological responses affecting oxygen delivery and their applications to human health. J. Physiol. doi: 10.1113/jp289666 42028867 PMC13120727

[B45] Fujisaka S. (2021). The role of adipose tissue M1/M2 macrophages in type 2 diabetes mellitus. Diabetol. Int. 12, 74–79. doi: 10.1007/s13340-020-00482-2 33479582 PMC7790922

[B46] Genchi V. A. D'Oria R. Palma G. Caccioppoli C. Cignarelli A. Natalicchio A. . (2021). Impaired leptin signalling in obesity: Is leptin a new thermolipokine? Int. J. Mol. Sci. 22, 6445. doi: 10.3390/ijms22126445 34208585 PMC8235268

[B47] Glazachev O. S. Kopylov F. Y. Susta D. Dudnik E. N. Zagaynaya E. (2017). Adaptations following an intermittent hypoxia-hyperoxia training in coronary artery disease patients: A controlled study. Clin. Cardiol. 40, 370–376. doi: 10.1002/clc.22670 28323322 PMC6490434

[B48] Glazachev O. S. Kryzhanovskaya S. Y. Zapara M. A. Dudnik E. N. Samartseva V. G. Susta D. (2021). Safety and efficacy of intermittent hypoxia conditioning as a new rehabilitation/secondary prevention strategy for patients with cardiovascular diseases: A systematic review and meta-analysis. Curr. Cardiol. Rev. 17, 88–99. doi: 10.2174/1573403X17666210514005235 33992064 PMC8950503

[B49] Gonzalez F. J. Xie C. Jiang C. (2018). The role of hypoxia-inducible factors in metabolic diseases. Nat. Rev. Endocrinol. 15, 21–32. doi: 10.1038/s41574-018-0096-z 30275460 PMC6624429

[B50] González-Domínguez Á. Visiedo-García F. M. Domínguez-Riscart J. González-Domínguez R. Mateos R. M. Lechuga-Sancho A. M. (2020). Iron metabolism in obesity and metabolic syndrome. Int. J. Mol. Sci. 21, 5529. doi: 10.3390/ijms21155529 32752277 PMC7432525

[B51] Goodpaster B. H. Sparks L. M. (2017). Metabolic flexibility in health and disease. Cell Metab. 25, 1027–1036. doi: 10.1016/j.cmet.2017.04.015 28467922 PMC5513193

[B52] Hai Y. Ren K. Zhang Y. Yang L. Cao Y. Yuan H. . (2024). HIF-1α serves as a co-linker between AD and T2DM. Biomed. Pharmacother. 171, 116158. doi: 10.1016/j.biopha.2024.116158 38242039

[B53] Heike M. (2009). Leptin-signaling pathways and leptin resistance. Forum Nutr. 63, 123–132. doi: 10.1159/000264400 19955780 PMC11129273

[B54] Hu X. Brown J. Nasiri A. Li Z. Wang H. Cartee G. D. . (2025). “ High-fat diet ablates an insulin-responsive pool of GLUT4 glucose transporters in skeletal muscle,” in Biorxiv. doi: 10.1101/2025.06.29.662135

[B55] Hyun-Yoo J. Miyong Y. Jaemin L. Eun-Ran P. Hyun-Jin K. Seon Rang W. . (2015). SIRT1 deacetylates and stabilizes hypoxia-inducible factor-1α (HIF-1α) via direct interactions during hypoxia. Biochem. Biophys. Res. Commun. 462, 294–300. doi: 10.1016/j.bbrc.2015.04.119 25979359

[B56] Ignacio M. David C. Elisa A. Garoa S. Teresa C. Gines V. . (2018). Additive effects of intermittent hypobaric hypoxia and endurance training on bodyweight, food intake, and oxygen consumption in rats. High Alt. Med. Biol. 19, 278–285. doi: 10.1089/ham.2018.0013 29957064

[B57] Inostroza-Mondaca F. Valdés D. Ramirez-Campillo R. Granacher U. (2025). Muscle strength, muscle morphology, and oxidative capacity in normal weight versus overweight and obese youth: A systematic review with meta-analysis. Sci. Rep. 15, 36108. doi: 10.1038/s41598-025-24024-5 41094088 PMC12528735

[B58] Isabel B.-P. Lisa R. Alfredo J. (2012). Oxidative stress and inflammation interactions in human obesity. J. Physiol. Biochem. 68, 297–306. doi: 10.1007/s13105-012-0154-2 22351038

[B59] Jebeile H. Kelly A. S. O'Malley G. Baur L. A. (2022). Obesity in children and adolescents: Epidemiology, causes, assessment, and management. Lancet Diabetes Endocrinol. 10, 351–365. doi: 10.1016/S2213-8587(22)00047-X 35248172 PMC9831747

[B60] Jensen M. D. Ryan D. H. Apovian C. M. Ard J. D. Comuzzie A. G. Donato K. A. . (2013). 2013 AHA/ACC/TOS guideline for the management of overweight and obesity in adults: A report of the American College of Cardiology/American Heart Association Task Force on Practice Guidelines and the Obesity Society. Circulation 129, S102. doi: 10.1161/01.cir.0000437739.71477.ee 24222017 PMC5819889

[B61] Jiali D. Ruohan Z. Fei L. Dan L. Cheng L. Ligen L. (2022). Sirtuins: key players in obesity-associated adipose tissue remodeling. Front. Immunol. 13, 1068986. doi: 10.3389/fimmu.2022.1068986 36505468 PMC9730827

[B62] Jiao X. Liu M. Li R. Li J. Wang L. Niu G. . (2024). Helpful to live healthier? Intermittent hypoxic/ischemic training benefits vascular homeostasis and lipid metabolism with activating SIRT1 pathways in overweight/obese individuals. Obes. Facts 17, 131–144. doi: 10.1159/000536093 38185107 PMC10987187

[B63] Jun L. Tao W. Geetha T. Babu J. (2024). Mitochondrial adaptation in skeletal muscle: Impact of obesity, caloric restriction, and dietary compounds. Curr. Nutr. Rep. 13, 500–515. doi: 10.1007/s13668-024-00555-7 38976215 PMC11327216

[B64] Kambič T. Debevec T. Lainscak M. (2025). Can intermittent hypoxic conditioning enhance the benefits of standard long COVID-19 rehabilitation? J. Cachexia Sarcopenia Muscle 16, e13769. doi: 10.1002/jcsm.13769 40034074 PMC11876854

[B65] Kawai T. Autieri M. V. Scalia R. (2021). Adipose tissue inflammation and metabolic dysfunction in obesity. Am. J. Physiol. Cell Physiol. 320, C375–C391. doi: 10.1152/ajpcell.00379.2020 33356944 PMC8294624

[B66] Kheniser K. Saxon D. R. Kashyap S. R. (2021). Long-term weight loss strategies for obesity. J. Clin. Endocrinol. Metab. 106, 1854–1866. doi: 10.1210/clinem/dgab091 33595666 PMC8427732

[B67] Knut M. Lars K. Natalia R. Sophie K. Anja M. Thomas B. . (2019). Hypoxia and exercise interactions on skeletal muscle insulin sensitivity in obese subjects with metabolic syndrome: Results of a randomized controlled trial. Int. J. Obes. (Lond) 44, 1119–1128. doi: 10.1038/s41366-019-0504-z 31819201

[B68] Kong J. Yang S. Nie Z. Zhang Y. Zuo Y. Zhang X. . (2025). Obesity: Pathophysiology and therapeutic interventions. Mol. Biomed. 6, 25. doi: 10.1186/s43556-025-00264-9 40278960 PMC12031720

[B69] Koskinas K. C. Van Craenenbroeck E. M. Antoniades C. Blüher M. Gorter T. M. Hanssen H. . (2024). Obesity and cardiovascular disease: An ESC clinical consensus statement. Eur. Heart J. 45, 4063–4098. doi: 10.1093/eurjpc/zwae279 39210706

[B70] Krishnan P. David C. Mark S. (2006). Altered respiratory physiology in obesity. Can. Respir. J. 13, 203–210. doi: 10.1155/2006/834786 16779465 PMC2683280

[B71] Lempesis I. Tsilingiris D. Liu J. Dalamaga M. (2022). Of mice and men: Considerations on adipose tissue physiology in animal models of obesity and human studies. Metab. Open 15, 100208. doi: 10.1016/j.metop.2022.100208 36092796 PMC9460138

[B72] Lempesis I. van Meijel R. Manolopoulos K. Goossens G. H. (2020). Oxygenation of adipose tissue: A human perspective. Acta Physiol. (Oxf) 228, e13298. doi: 10.1111/apha.13298 31077538 PMC6916558

[B73] Li-Ling C. Shih-Wei C. Yu-Min C. Hsin-Yi L. John L. Desmond C. . (2004). Effect of prolonged intermittent hypoxia and exercise training on glucose tolerance and muscle GLUT4 protein expression in rats. J. Biomed. Sci. 11, 838–846. doi: 10.1007/bf02254369 15591781

[B74] Liam P. Fabien A. (2017). Acute normobaric hypoxia increases post-exercise lipid oxidation in healthy males. Front. Physiol. 8, 293. doi: 10.3389/fphys.2017.00293 28567018 PMC5434119

[B75] Ling Q. Sailan W. Ran J. Zhi S. Cen L. Yang X. . (2008). The effect of intermittent hypoxia on bodyweight, serum glucose and cholesterol in obesity mice. Pak. J. Biol. Sci. 11, 869–875. doi: 10.3923/pjbs.2008.869.875 18814648

[B76] Lv R. Liu Z. Zhang X. Dong Y. Wang Y. He Y. . (2023). Pathophysiological mechanisms and therapeutic approaches in obstructive sleep apnea syndrome. Signal. Transduct Target Ther. 8, 218. doi: 10.1038/s41392-023-01496-3 37230968 PMC10211313

[B77] Mallet R. T. Manukhina E. B. Ruelas S. S. Caffrey J. L. Downey H. F. (2018). Cardioprotection by intermittent hypoxia conditioning: Evidence, mechanisms, and therapeutic potential. Am. J. Physiol. Heart Circ. Physiol. 315, H216–H232. doi: 10.1152/ajpheart.00060 29652543 PMC6139623

[B78] Michael D. Hans-Joachim S. (2012). Impact of obesity on exercise performance and pulmonary rehabilitation. Respirology 17, 899–907. doi: 10.1111/j.1440-1843.2012.02151.x 22348704

[B79] Navarrete-Opazo A. Mitchell G. S. (2014). Therapeutic potential of intermittent hypoxia: A matter of dose. Am. J. Physiol. Regul. Integr. Comp. Physiol. 307, R1181–R1197. doi: 10.1152/ajpregu.00208.2014 25231353 PMC4315448

[B80] Nicholas R. Rosalba C. (2016). A case of remission from pre-diabetes following intermittent hypoxic training. Obes. Res. Clin. Pract. 10, 487–491. doi: 10.1016/j.orcp.2016.05.008 27312533

[B81] Nicolucci A. Maffeis C. (2022). The adolescent with obesity: What perspectives for treatment? Ital. J. Pediatr. 48, 9. doi: 10.1186/s13052-022-01205-w 35033162 PMC8761267

[B82] Noria S. F. Shelby R. D. Atkins K. D. Nguyen N. T. Gadde K. M. (2023). Weight regain after bariatric surgery: Scope of the problem, causes, prevention, and treatment. Curr. Diabetes Rep. 23, 31–42. doi: 10.1007/s11892-023-01498-z 36752995 PMC9906605

[B83] Obradovic M. Sudar-Milovanovic E. Soskic S. Essack M. Arya S. Stewart A. J. . (2021). Leptin and obesity: Role and clinical implication. Front. Endocrinol. (Lausanne) 12, 585887. doi: 10.3389/fendo.2021.585887 34084149 PMC8167040

[B84] Olateju A. Opaleye-Enakhimion T. Udeogu J. Asuquo E. Olaleye K. Osa E. . (2023). A systematic review on the effectiveness of diet and exercise in the management of obesity. Diabetes Metab. Syndr. 17, 102759. doi: 10.1016/j.dsx.2023.102759 37084486

[B85] Omair A. Travis J. (2020). Obesity-related hypertension: a review of pathophysiology, management, and the role of metabolic surgery. Gland Surg. 9, 80–93. doi: 10.21037/gs.2019.12.03 32206601 PMC7082272

[B86] P G.-M. A L.-P. J A. A L. M P. F A. . (2015). Impact of intermittent hypoxia and exercise on blood pressure and metabolic features from obese subjects suffering sleep apnea-hypopnea syndrome. J. Physiol. Biochem. 71, 589–599. doi: 10.1007/s13105-015-0410-3 25913417

[B87] Perdomo C. M. Cohen R. V. Sumithran P. Clément K. Frühbeck G. (2023). Contemporary medical, device, and surgical therapies for obesity in adults. Lancet 401, 1116–1130. doi: 10.1016/s0140-6736(22)02403-5 36774932

[B88] Peters U. Dixon A. E. Forno E. (2018). Obesity and asthma. J. Allergy Clin. Immunol. 141, 1169–1179. doi: 10.1016/j.jaci.2018.02.004 29627041 PMC5973542

[B89] Piché M. E. Tchernof A. Després J. P. (2020). Obesity phenotypes, diabetes, and cardiovascular diseases. Circ. Res. 126, 1477–1500. doi: 10.1161/CIRCRESAHA.120.316101 32437302

[B90] Piester K. Jagtap N. Kalapala R. (2023). Review of paediatric obesity and non-alcoholic fatty liver disease—a focus on emerging non-pharmacologic treatment strategies. Pediatr. Obes. 18, e13067. doi: 10.1111/ijpo.13067 37602954

[B91] Puri S. Panza G. Mateika J. (2021). A comprehensive review of respiratory, autonomic and cardiovascular responses to intermittent hypoxia in humans. Exp. Neurol. 341, 113709. doi: 10.1016/j.expneurol.2021.113709 33781731 PMC8527806

[B92] Qiu Y. Fernández-García B. Lehmann H. I. Li G. Kroemer G. López-Otín C. . (2023). Exercise sustains the hallmarks of health. J. Sport Health Sci. 12, 8–35. doi: 10.1016/j.jshs.2022.10.003 36374766 PMC9923435

[B93] Qiu Y. Wu Y. Yang H. Zhang H. Liu X. Sun H. . (2022). The role of iron metabolism in chronic diseases related to obesity. Mol. Med. 28, 130. doi: 10.1186/s10020-022-00558-6 36335331 PMC9636637

[B94] Quiñones M. Martínez-Grobas E. Fernø J. Pérez-Lois R. Seoane L. M. Al Massadi O. (2021). Hypothalamic actions of SIRT1 and SIRT6 on energy balance. Int. J. Mol. Sci. 22, 1430. doi: 10.3390/ijms22031430 33572672 PMC7866978

[B95] Richard M. Neil M. Paul H. Gary C. Peter W. (2011). Acute hypoxia and exercise improve insulin sensitivity (S(I) (2*)) in individuals with type 2 diabetes. Diabetes Metab. Res. Rev. 27, 94–101. doi: 10.1002/dmrr.1156 21218513

[B96] Rocco B. Gianluca T. Maurizio C. Enzo N. (2018). Insulin resistance in obesity: an overview of fundamental alterations. Eat Weight Disord. 23, 149–157. doi: 10.1007/s40519-018-0481-6 29397563

[B97] Rouatbi S. Ghannouchi I. Kammoun R. Ben Saad H. (2020). The ventilatory and diffusion dysfunctions in obese patients with and without obstructive sleep apnea-hypopnea syndrome. J. Obes. 2020, 8075482. doi: 10.1155/2020/8075482 32104601 PMC7035560

[B98] Ruban A. Stoenchev K. Ashrafian H. Teare J. (2019). Current treatments for obesity. Clin. Med. (Lond) 19, 205–212. doi: 10.7861/clinmedicine.19-3-205 31092512 PMC6542229

[B99] Serebrovskaya T. V. Manukhina E. B. Smith M. L. Downey H. F. Mallet R. T. (2008). Intermittent hypoxia: Cause of or therapy for systemic hypertension? Exp. Biol. Med. (Maywood) 233, 627–650. doi: 10.3181/0710-mr-267 18408145

[B100] Serebrovskaya T. V. Xi L. (2016). Intermittent hypoxia training as non-pharmacologic therapy for cardiovascular diseases: Practical analysis on methods and equipment. Exp. Biol. Med. (Maywood) 241, 1708–1723. doi: 10.1177/1535370216657614 27407098 PMC4999622

[B101] Seth M. Biswas R. Ganguly S. Chakrabarti N. Chaudhuri A. G. (2020). Leptin and obesity. Physiol. Int. 107, 455–468. doi: 10.1556/2060.2020.00038 33355539

[B102] Shah N. Kaltsakas G. (2023). Respiratory complications of obesity: From early changes to respiratory failure. Breathe (Sheff) 19, 220263. doi: 10.1183/20734735.0263-2022 37378063 PMC10292783

[B103] Shams E. Kamalumpundi V. Peterson J. Gismondi R. A. Oigman W. de Gusmão Correia M. L. (2022). Highlights of mechanisms and treatment of obesity-related hypertension. J. Hum. Hypertens. 36, 785–793. doi: 10.1038/s41371-021-00644-y 35001082

[B104] Shan L. Mingyang L. Lirun Z. Keyu C. Shiran W. Xinde L. . (2023). SIRT1/SREBPs-mediated regulation of lipid metabolism. Pharmacol. Res. 199, 107037. doi: 10.1016/j.phrs.2023.107037 38070792

[B105] Szabo L. McCracken C. Cooper J. Rider O. J. Vago H. Merkely B. . (2023). The role of obesity-related cardiovascular remodelling in mediating incident cardiovascular outcomes: a population-based observational study. Eur. Heart J. Cardiovasc. Imaging 24, 921–929. doi: 10.1093/ehjci/jeac270 36660920 PMC10284050

[B106] T V. S. Z O. S. E E. K. (2016). Fitness and therapeutic potential of intermittent hypoxia training: A matter of dose. Fiziol Zh. 62, 78. doi: 10.15407/fz62.03.078 29569889

[B107] Tak Y. J. Lee S. Y. (2021). Long-term efficacy and safety of anti-obesity treatment: Where do we stand? Curr. Obes. Rep. 10, 14–30. doi: 10.1007/s13679-020-00422-w 33410104 PMC7787121

[B108] Tee J. Cooke M. Chong M. Yeo W. Camera D. (2023a). Mechanisms for combined hypoxic conditioning and divergent exercise modes to regulate inflammation, body composition, appetite, and blood glucose homeostasis in overweight and obese adults: A narrative review. Sports Med. 53, 327–348. doi: 10.1007/s40279-022-01782-0 36441492 PMC9877079

[B109] Tee J. Parr E. Cooke M. Chong M. Rahmat N. Md Razali N. . (2023b). Combined effects of exercise and different levels of acute hypoxic severity: a randomized crossover study on glucose regulation in adults with overweight. Front. Physiol. 14, 1174926. doi: 10.3389/fphys.2023.1174926 37123278 PMC10133678

[B110] Tsilingiris D. Tzeravini E. Koliaki C. Dalamaga M. Kokkinos A. (2021). The role of mitochondrial adaptation and metabolic flexibility in the pathophysiology of obesity and insulin resistance: An updated overview. Curr. Obes. Rep. 10, 191–213. doi: 10.1007/s13679-021-00434-0 33840072

[B111] van Gerwen J. Shun-Shion A. S. Fazakerley D. J. (2023). Insulin signalling and GLUT4 trafficking in insulin resistance. Biochem. Soc Trans. 51, 1057–1069. doi: 10.1042/bst20221066 37248992 PMC10317183

[B112] van Meijel R. Vogel M. Jocken J. Vliex L. Smeets J. Hoebers N. . (2021). Mild intermittent hypoxia exposure induces metabolic and molecular adaptations in men with obesity. Mol. Metab. 53, 101287. doi: 10.1016/j.molmet.2021.101287 34224918 PMC8355948

[B113] Vultur G. Grigorescu B. Huţanu A. Ianoşi E. Budin C. Jimborean G. (2025). A multidisciplinary approach to obesity hypoventilation syndrome: From diagnosis to long-term management—a narrative review. Diagnostics (Basel) 15, 2120. doi: 10.3390/diagnostics15172120 40941607 PMC12428154

[B114] Wadden T. A. Tronieri J. S. Butryn M. L. (2020). Lifestyle modification approaches for the treatment of obesity in adults. Am. Psychol. 75, 235. doi: 10.1037/amp0000517 32052997 PMC7027681

[B115] Wolfe B. M. Kvach E. Eckel R. H. (2016). Treatment of obesity: Weight loss and bariatric surgery. Circ. Res. 118, 1844–1855. doi: 10.1161/CIRCRESAHA.116.307591 27230645 PMC4888907

[B116] Xiong-Fei P. Limin W. An P. Epidemiology and determinants of obesity in China. Lancet Diabetes Endocrinol. 9, 373–392. doi: 10.1016/S2213-8587(21)00045-0 34022156

[B117] Xu Y. Zeng Y. Yan J. Xu J. (2021). Hypoxic exercise exacerbates hypoxemia and acute mountain sickness in obesity: A case analysis. Int. J. Environ. Res. Public Health 18, 9078. doi: 10.3390/ijerph18179078 34501667 PMC8430682

[B118] Xue L. Zou H. Ruan Z. Chen Y. Lai Y. Yao Z. . (2023). Pharmacoeconomic evaluation of anti-obesity drugs for chronic weight management: A systematic review of literature. Front. Endocrinol. (Lausanne) 14, 1254398. doi: 10.3389/fendo.2023.1254398 38027186 PMC10658190

[B119] Yanovski S. Z. Yanovski J. A. (2024). Approach to obesity treatment in primary care: A review. JAMA Intern. Med. 184, 818–829. doi: 10.1001/jamainternmed.2023.8526 38466272 PMC12182808

[B120] Yao M. Zhang L. Wang L. (2023). Astragaloside IV: A promising natural neuroprotective agent for neurological disorders. Biomed. Pharmacother. 159, 114229. doi: 10.1016/j.biopha.2023.114229 36652731

[B121] Yuan Y. Liu W. Gu J. Ji X. Nan G. (2022). Intermittent hypoxia conditioning as a potential prevention and treatment strategy for ischemic stroke: Current evidence and future directions. Front. Neurosci. 16, 1067411. doi: 10.3389/fnins.2022.1067411 36507357 PMC9732261

[B122] Zhang Y. Eugeny E. Leonid K. Li J. Chen X. Elena N. . (2024). Assessing the importance and safety of hypoxia conditioning for patients with occupational pulmonary diseases: A recent clinical perspective. Biomed. Pharmacother. 178, 117275. doi: 10.1016/j.biopha.2024.117275 39126774

[B123] Zhang X. Liu Y. Lu J. Cao Y. Zhu W. (2025). Compared to moderate-intensity continuous training, short-term high-intensity interval training demonstrates enhanced effects on metabolic flexibility in adult males with obesity. J. Exerc Sci. Fit 23, 370–378. doi: 10.1016/j.jesf.2025.07.005 40799940 PMC12341516

[B124] Zhang Q. Zhao Y. Li Y. Ding Y. Wang L. Ji X. (2023). Intermittent hypoxia conditioning: A potential multi-organ protective therapeutic strategy. Int. J. Med. Sci. 20, 1551–1561. doi: 10.7150/ijms.86622 37859700 PMC10583178

[B125] Zhaowei K. Yanpeng Z. Yang H. (2013). Normobaric hypoxia training causes more weight loss than normoxia training after a 4-week residential camp for obese young adults. Sleep Breath 18, 591–597. doi: 10.1007/s11325-013-0922-4 24318688

