# Supplementary material for: Intermittent hypoxic conditioning as a novel strategy for obesity treatment: current evidence and future perspectives
Source: Front Physiol. 2026 Jun 26;17:1842034. doi: 10.3389/fphys.2026.1842034 (PMC13349900; doi:10.3389/fphys.2026.1842034)
Supplement: Supplementary file 1 [file Supplementaryfile1.docx]

**Supplementary Materials**


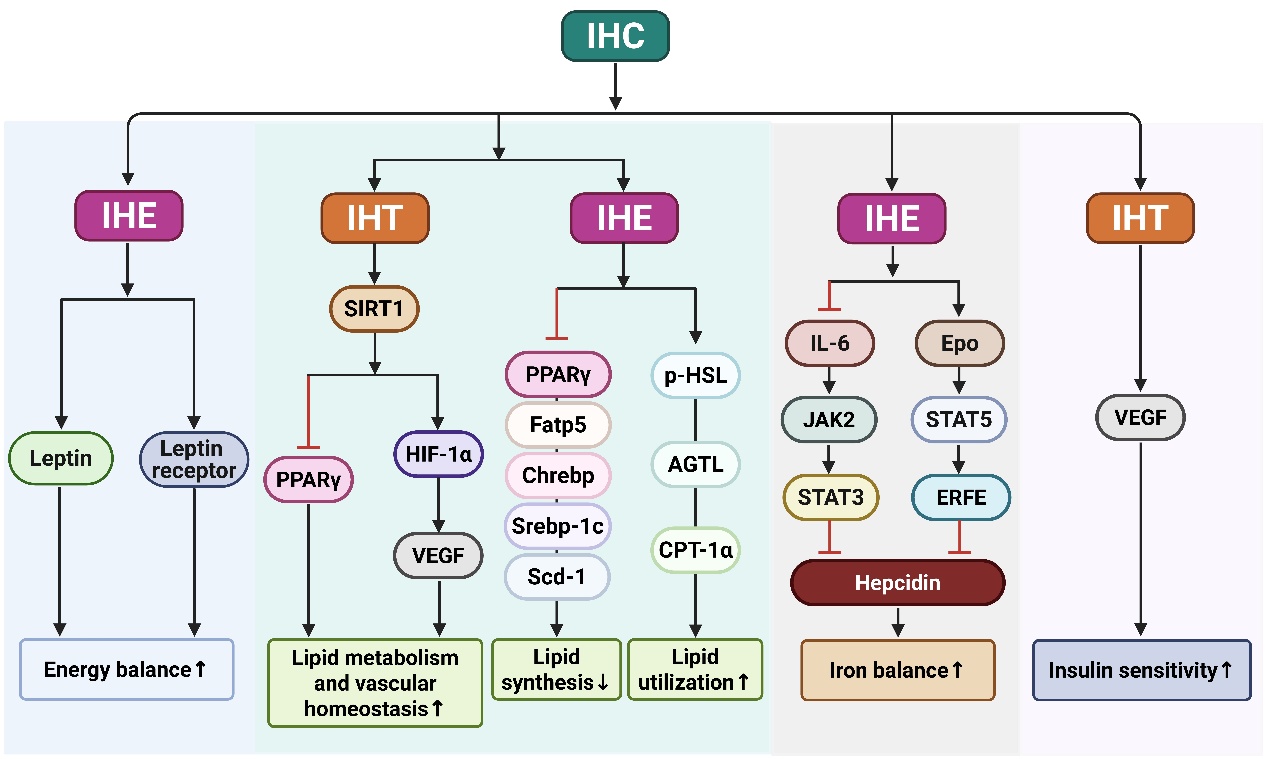


**Figure S1. Proposed mechanistic targets of IHC in obesity and its complications.** Schematic overview of the key pathways involved in the metabolic effects of IHC, including leptin-mediated energy balance, SIRT1/PPARγ and SIRT1/HIF-1α/VEGF-mediated lipid metabolism and vascular homeostasis, lipid enzyme-mediated hepatic lipid handling, IL-6/JAK2/STAT3 and Epo/STAT5/ERFE-mediated iron metabolism, and VEGF-mediated glucose metabolism.

Abbreviations: IHC: intermittent hypoxic conditioning; IHE: intermittent hypoxic exposure; IHT: intermittent hypoxic training; SIRT1: Sirtuin 1; PPARγ: peroxisome proliferator-activated receptor-γ; Fatp5: fatty acid transport protein 5; Chrebp: carbohydrate response element-binding protein; Srebp-1c: sterol regulatory element-binding protein-1c; Scd-1: stearoyl-CoA desaturase-1; p-HSL: phosphorylated hormone-sensitive lipase; ATGL: adipose triglyceride lipase; CPT-1α: carnitine palmitoyl transferase-1α; IL-6: interleukin6; JAK2: Janus kinase 2; STAT3: signal transducer and activation of the transcription 3; Epo: erythropoietin; STAT5: signal transducer and activation of the transcription 5; ERFE: erythroferrone; HIF-1α: hypoxia-inducible factor-1α; VEGF: vascular endothelial growth factor.
